# Activity-induced interactions and cooperation of artificial microswimmers in one-dimensional environments

**DOI:** 10.1038/s41467-022-29430-1

**Published:** 2022-04-01

**Authors:** Stefania Ketzetzi, Melissa Rinaldin, Pim Dröge, Joost de Graaf, Daniela J. Kraft

**Affiliations:** 1 Soft Matter Physics, Huygens-Kamerlingh Onnes Laboratory, Leiden University, P.O. Box 9504, 2300 RA Leiden, The Netherlands; 2 Institute for Theoretical Physics, Center for Extreme Matter and Emergent Phenomena, Utrecht University, Princetonplein 5, 3584 CC Utrecht, The Netherlands; 3 Present Address: Max Planck Institute of Molecular Cell Biology and Genetics, Pfotenhauerstr. 108, 01307 Dresden, Germany

**Keywords:** Soft materials, Colloids, Self-assembly

## Abstract

Cooperative motion in biological microswimmers is crucial for their survival as it facilitates adhesion to surfaces, formation of hierarchical colonies, efficient motion, and enhanced access to nutrients. Here, we confine synthetic, catalytic microswimmers along one-dimensional paths and demonstrate that they too show a variety of cooperative behaviours. We find that their speed increases with the number of swimmers, and that the activity induces a preferred distance between swimmers. Using a minimal model, we ascribe this behavior to an effective activity-induced potential that stems from a competition between chemical and hydrodynamic coupling. These interactions further induce active self-assembly into trains where swimmers move at a well-separated, stable distance with respect to each other, as well as compact chains that can elongate, break-up, become immobilized and remobilized. We identify the crucial role that environment morphology and swimmer directionality play on these highly dynamic chain behaviors. These activity-induced interactions open the door toward exploiting cooperation for increasing the efficiency of microswimmer motion, with temporal and spatial control, thereby enabling them to perform intricate tasks inside complex environments.

## Abstract (web summary)

Biological microswimmers such as bacteria show collective motion that is made possible by an intricate interplay of sensing and signaling. Ketzetzi et al. reproduce this phenomenon in a catalytic system undergoing, for instance, cooperative speed-ups and dynamic reconfiguration of microswimmer chains.

## Introduction

Many microorganisms crucially rely on cooperation for their survival and thriving. Cooperation greatly enhances microorganism motility and overall motion efficiency^1^, and often leads to the formation of organized, complex colonies. For example, spermatozoa self-assemble into train-like structures to enhance fertilization^2^, *Volvox* algae form colonies to propel and facilitate fluid flows with nutrients and chemical messengers^3^, and cancer cells secrete chemicals to communicate and promote tumor growth^4^. Similarly, bacteria cooperate to enhance surface adhesion during biofilm formation^5^, which increases their resistance to environmental stresses and drugs, their spreading, and the efficiency of nutrient capture^5–7^. At high densities, bacterial colonies again rely on cooperation to form swarms with large-scale dynamic patterns, such as whirls and jets, to expand and to explore their surroundings while simultaneously reducing their competition for nutrients^8,9^. These vital behaviors are achieved by exploiting interactions based on hydrodynamic and steric effects^10^, as well as chemical signaling, which lead to quorum-sensing when it regulates density-dependent gene expression^11^.

Similar to their biological counterparts, synthetic swimmers also exhibit directed motion inside liquid environments^12^, even under real-world conditions, i.e., inside patterned^13–23^ and biological environments^24–26^. Achieving precise motion control in living organisms and lab-on-a-chip devices^27,28^ offers exciting opportunities for realizing technologically and bio-medically relevant applications^12^. For example, swimmers could be deployed to perform in vivo drug delivery^29,30^ inside complex and crowded environments^31,32^. Drawing inspiration from biological systems and their efficiency-increasing strategies, it is desirable that tasks are performed not only on the single- but also on the multi-swimmer level^33,34^. For instance, if employed in drug delivery, collections of swimmers may reach the desired target faster, or deliver a higher dosage^35^. Cooperative behavior and communication between the microswimmers could furthermore enable different types of delivery, in which for example dosages are applied at specific times or time intervals^36^.

Although collective effects, such as enhanced aggregation, cluster and crystal formation, ordering and phase separation, have been observed for synthetic systems in two^37–39^ and three dimensions^40,41^ (2D and 3D, respectively), these effects can in principle be explained by volume exclusion and persistent motion of the swimmer^1,38,40^. That is, they do not require cooperation, which typically relies on information exchange to enhance the efficiency of their behavior. Even the exciting recently observed corralling of passive particles by swarms of light-driven synthetic swimmers was explained purely by geometric arguments^42^. Other collective effects such as the formation of self-spinning microgears^43^ and active colloidal molecules^44^ required external fields for their assembly and/or propulsion. Thus, while cooperation is a type of collective effect, the inverse is not necessarily true: the collective behavior of synthetic microswimmers observed so far at higher densities did not signify that they collaborate and cooperate in the same sense as biological swimmers, which employ signaling and sensing.

Here, we demonstrate that catalytically propelled model microswimmers exhibit a wealth of phenomena due to activity-induced interactions along closed one-dimensional (1D) paths. Single swimmers move with fixed speed along paths of constant curvature, independent of the value of the curvature. We further find that multiple swimmers moving along the same path exhibit a cooperative speedup, i.e., their speed increases with the number of swimmers. Simultaneously, their activity induces a preferred and unexpectedly large separation between them. We provide a simple model that reveals that long-range swimmer cooperation can originate from a combination of hydrodynamic and chemical couplings. Our model shows qualitative agreement with the experiment using only a few, physically motivated choices for the fit parameters. For more compact configurations of swimmers, which we termed chains, we experimentally demonstrate rich locomotion behavior induced by chain fission and fusion, which has only been considered theoretically in the context of magnetic swimmers^45^. Lastly, we reveal that chain formation and breakup can be tuned using the change in the curvature of the local path.

## Results

### Catalytic microswimmer motion in one-dimensional environments

**Fig. 1:**
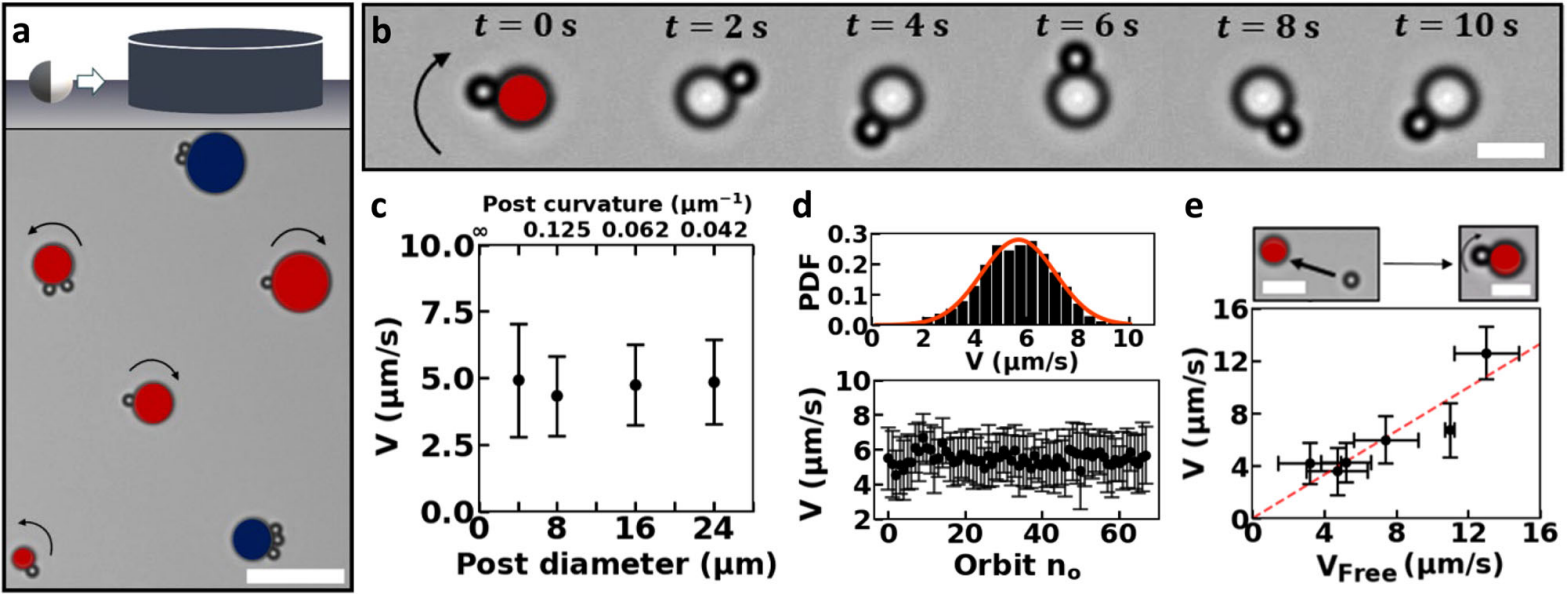
Catalytic microswimmer motion along circular posts: single-particle orbiting. **a** Schematic of the experimental setup (top) and light microscopy image (bottom) of the corresponding experiment: catalytic microswimmers orbit circular 3D posts, microprinted on planar substrates. Scale bar is 20 μm. Coloring is used to distinguish the stationary posts from our swimmers, and indicates whether the attached swimmers were mobile (red posts) with the arrow denoting their direction in orbit, or immobile (blue posts) due to the swimmers initially orbiting toward opposing directions. **b** Time series of light microscopy images of a (2.00 ± 0.05) μm diameter swimmer orbiting a 4 μm diameter post, with the arrow denoting its constant direction of motion. Scale bar is 5 μm. **c** Propulsion speed along the post as a function of post diameter. All data were taken at otherwise fixed experimental conditions. **d** Top: propulsion speed of an individual swimmer in orbit follows a Gaussian distribution. Measurement duration was 4 min. Bottom: same propulsion speed data plotted as a function of number of orbits. **e** Propulsion speed for the swimmers before orbiting, i.e., free speed on the planar substrate, plotted against their speed in orbit. The dashed line is a least-squares fit with *y* = *ax* and *a* = 0.83 ± 0.08, in line with Ref. ^14^.

To study their dynamic behavior and interactions, we confine swimmers to 1D tracks by exploiting their strong affinity for surfaces^15,16,19,46,47^. This affinity stems from their propulsion mechanism^15,16^, which is based on an asymmetric catalytic decomposition of H_2_O_2_ on their Pt-coated hemisphere^48^. We equip planar substrates with designed 3D microprinted posts, thereby effectively creating preferred 1D environments around the posts, where the swimmers can be in close proximity to both posts and substrates^15,16,19^. Figure 1a shows one example of our experimental setups featuring circular posts. Here, the H_2_O_2_ decomposition reaction sets up gradients in solute molecule concentration. These act over the swimmer surfaces, posts, and substrates, inducing phoretic and osmotic flows, which in turn cause self-propulsion^48^ and swimmer capture^16,46,47^, though the exact details of how these behaviors come about remain unclear. Similar to previous work^16,19^, we observed that when a swimmer encounters a post, it quickly gets captured into motion along it, and is retained there for very long times.

Once attached to a circular post, swimmers with diameters of (2.00 ± 0.05) μm and Pt coating thicknesses of (4.7 ± 0.2) nm moved with equal probability in either the clockwise or counterclockwise direction, without switching direction. Single swimmers, as well as multiple swimmers with the same direction of motion, on a given post, orbited their posts with approximately constant speed. We highlighted these swimmers by coloring their corresponding posts in red in Fig. 1a and indicated their direction of motion with an arrow. These swimmers continued their orbiting motion for at least 30 min in our experiment, longer than the ones in Ref. ^16^ which orbited for ≈1 min, and longer than swimmers trapped around spheres which showed hopping rates of about 10^−3^ Hz at similar hydrogen peroxide concentration^19^. However, swimmers that moved in opposing directions on a given post hindered each other’s motion after the collision, leading to an immobilized state, similar to the collision dynamics along a straight edge^15^. An example of the immobilized state can be seen in Supplementary Movie 1. We indicated these immobilized clusters by coloring their adjacent posts blue in Fig. 1a.

Surprisingly, long-term capture around posts happens even when the post diameter is comparable to the swimmer size, see for example Fig. 1b which shows a 2 μm diameter swimmer orbiting a 4 μm diameter post, a size ratio that is much smaller than those considered in Ref. ^16^. The capture around such small posts is in stark contrast with simulations on model squirmers^49,50^, which are often used to approximate synthetic swimmers such as the ones used in our experiments. For model squirmers, capture has only been proposed for posts of sizes several times larger than the squirmer^49^. Moreover, simulations for the orbiting of a squirmer around a sphere predict a relation between curvature and squirmer speed, as can be readily obtained from the data in Ref. ^50^. To test these predictions, we track the swimmers in time and extract their speed in orbit using python routines^51^. We find the same self-propulsion speed irrespective of the value of the (constant) curvature of our posts, at least for the range of curvatures considered in Fig. 1c. These discrepancies between our experiments and simulations imply that catalytic swimmers are different from pure squirmers. We hypothesize that the origin of this difference can be traced to their propulsion mechanism and the long-range solute gradients that act across the substrate and posts^46,47^.

Despite orbiting short tracks, we find that propulsion speed in orbit is quite stable, see Fig. 1d, which shows that speed of a single swimmer in orbit follows a Gaussian distribution with a narrow width. Note that the presence of the post itself does not have a considerable effect on propulsion speed: the speed of a swimmer in orbit is only slightly reduced with respect to its free speed parallel to the substrate far away from the posts, see Fig. 1e. The dashed line in Fig. 1e is a least-squares fit with *y* = *ax* where *a* is (0.83 ± 0.08), a value that is in line with the slope obtained previously for bimetallic microrods^14^. These findings indicate that any hydrodynamic and/or phoretic coupling to the post leads to a subdominant contribution to the speed. Presumably, this is because of the low post heights (8.0 ± 0.2 μm).

### Cooperative motion between microswimmers

**Fig. 2:**
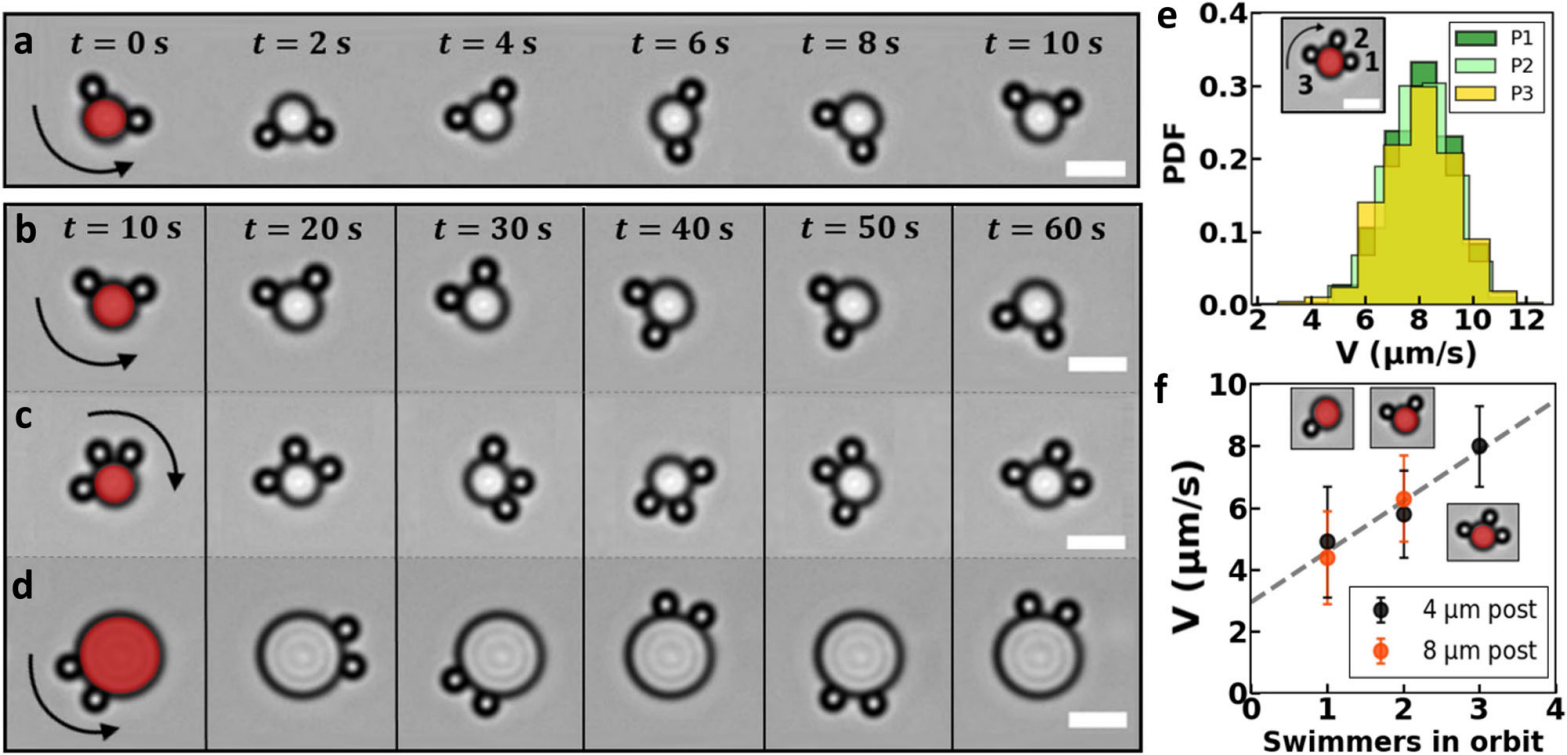
Multiple microswimmers cooperatively orbit circular posts. Red color has been added to distinguish the stationary posts from the orbiting swimmers. Scale bars are 5 μm. Sequence of light microscopy images showing orbiting of **a**, **b** two and **c** three swimmers around a 4 μm diameter post, and **d** two swimmers around an 8 μm diameter post. See also Supplementary Movies 2–4. Each column in panels **b**–**d** shares the same timestamp, indicated in **b**. **e** The probability density functions (PDFs) of propulsion speed in orbit for three swimmers on a post are almost identical. **f** Average propulsion speed as a function of the number of swimmers in orbit for two post sizes, showing that speed increases with the number of comoving swimmers for both post sizes in a similar fashion. All measurements were taken under the same experimental conditions. The dashed line is a least-squares fit with *y* = *αx* + *b*, with *α* and *b* being (1.6 ± 0.2) μm/s and (2.9 ± 0.4) μm/s, respectively.

Intriguing effects occur when multiple swimmers orbit the same post: the swimmers move with similar speeds while also maintaining comparable distances, see Fig. 2 and Supplementary Movies 2–4. We will refer to this well-separated collective of swimmers as a train. The observed constancy in speed and distance appears both for two (Fig. 2a, b) and three comoving swimmers (Fig. 2c), and is independent of the post curvature (Fig. 2d). More quantitatively, we find that all swimmers orbiting the same post, in fact, have almost the same speed distribution, independent of the particle number and post size. See Fig. 2e for the speed distributions of three swimmers on a 4 μm post and Supplementary Fig. S1 for additional data.

Strikingly, however, we found that speed increases with the number of co-orbiting swimmers, as shown in Fig. 2f for posts with 4 and 8 μm diameters. That is, two particles orbit faster than one, and in turn three particles orbit faster than two. Under otherwise fixed conditions, their speed increases by ≈20% and 60% for two and three comoving swimmers on 4 μm posts, respectively, and by ≈40% for two comoving swimmers on the 8 μm post, in comparison to single swimmers. Theoretically, a speedup can be captured with a minimal model, as we will show later. We also note that while we never observed four comoving particles on these small posts, we do not exclude that this is possible. We instead attribute this observation to both the small post size and the significant probability (92%) that one or more of the four swimmers moved in the opposing direction. Interestingly, for our active system there is no significant speedup of a pair of particles with respect to a separated third particle moving along the post. This contrasts strongly with the result of passive, driven particles in a toroidal optical trap^52–54^, where pair formation and breaking is observed. In that case, two driven particles overtake a third, which then leads to a fracture of the triplet with the two lead particles moving off. The driven-particle result can be understood using hydrodynamic theory^52–54^. Clearly, our self-propelled system shows greater stability, which we will return to shortly.

The above findings strongly suggest cooperative motion of the microswimmers: swimmers interact in the near field via chemical gradients and (associated) hydrodynamic flows, leading to a multi-bound state that exhibits a collective speedup. This speedup is independent of the post size for the here considered 4 and 8 μm posts, see Fig. 2f. The dashed line represents a least-squares fit with *y* = *αx* + *b*, with *α* (1.6 ± 0.2) μm/s and *b* (2.9 ± 0.4) μm/s, implying a linear relationship between the number of swimmers and their collective speed. This observation of a speedup, as well as the seemingly constant swimmer distance in Fig. 2, is surprising. To achieve the former, swimmers must experience a coupling that adjusts their speed.

### Quantification of microswimmer interactions

**Fig. 3:**
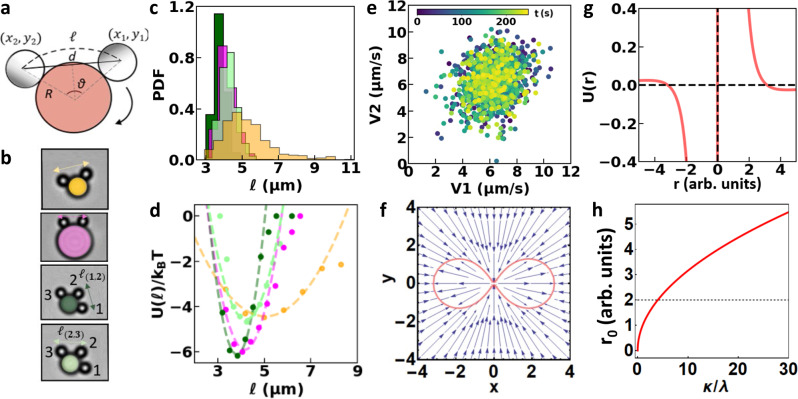
Interactions of microswimmers comoving along circular posts: experiment and modeling. **a** Representation of the arc distance for two swimmers orbiting in the clockwise direction away from their Pt-coated shaded caps. **b** Snapshots of swimmer pairs in orbit, color-coded so that each color marks the corresponding swimmer pair in panels **c** and **d**. **c** PDF of the arc distance between comoving swimmers, showing that swimmers assume a relatively constant distance in orbit with minimum center-to-center distance ≈3 μm. Measurement duration is ≈5 min. **d** Potential energy in units of thermal energy, as obtained from the swimmer distances in **c** using the Boltzmann distribution. The dashed lines represent least-squares fits with $y=\frac{1}{2}k{(x-{x}_{0})}^{2}+{y}_{0}$. All fitted parameters are listed in the Supplementary Information (SI). **e** Scatter plot of the speeds of the two swimmers comoving along the 8 μm post colored magenta in panels **b**–**d**, showing that swimmer speeds are not correlated. **f**–**h** Effective separation between swimmers based on hydrodynamic and osmotic balance. **f** The balance between an inward osmotic flow along the wall and an outward pusher-type dipolar flow away from the swimmer leads to a curve of zero velocity depicted in red. **g** Two swimmers that lie head-to-tail as in panel (**a**) assume a fixed distance as evidenced by the *x*-axis intercept. The shape of the relative velocity generates an effective potential. **h** Swimmer separation distance as a function of the ratio between the osmotic and pusher contributions, with *λ* and *κ* indicating the respective strengths of the inward and outward flows. **f**–**h** See Supplementary Information for details and the used code, together with an in-depth analysis that additionally accounts for the asymmetric Pt cap and swimmer trains of up to six swimmers.

To understand the origin of the coupling, we quantify the swimmer separation via the arc length, *ℓ*, between comoving swimmers as depicted in Fig. 3a. We measure the distances between various swimmer pairs, on differently sized posts and with different number of attached swimmers, see Fig. 3b. In all cases, we find that swimmers orbit the posts at a preferred distance (Fig. 3c), in line with our expectations based on Fig. 2. We notice that swimmers never approach closer than a minimum center-to-center distance of (3.0 ± 0.1) μm. In addition, the arc distances show similar distributions, albeit with slightly different peak values and widths.

These common features raise the question whether the constancy in the distance stems from correlations between the speeds of the comoving swimmers. However, a closer examination reveals that speeds always vary independently of one another, see the scatter plot of the speeds of two comoving swimmers along an 8 μm post, *V*_1_(*t*) vs. *V*_2_(*t*), where *t* is time in Fig. 3e and Supplementary Fig. S2. Already, the symmetric shape of the scatter plot indicates that they are not correlated, which is further supported by a Pearson correlation coefficient, see Methods, of 0.2. In addition, we excluded time-delayed correlations by considering the correlation between *V*_1_(*t*) and *V*_2_(*t* + *τ*) see SI, with *τ* the time between two frames. Again, the Pearson correlation coefficient was 0.1, signifying no speed correlation. Hence, there must be an alternative mechanism that can explain why swimmers move with the same average speed and stable distance.

The random fluctuations around a well-defined average distance suggest that an effective potential can be fitted. This effective potential must be induced by the active state of both swimmers. A single active particle is not sufficient, because passive particles in the vicinity of an active particle do not become confined, but are instead either dragged along the fluid flow around the active particle^55^, or attracted to the active particle site^39^. We fit the relative potential *U*(*ℓ*) using the Boltzmann distribution, ${{{{{{{\rm{PDF}}}}}}}}(\ell )=\exp (-U(\ell )/{k}_{B}T)$, with *k*_*B*_ the Boltzmann constant and *T* the temperature as follows. We consider the motion in the rotating frame of reference that comoves with the average displacement of the swimmers. Note that this is a non-inertial frame of reference for an out-of-equilibrium state, yet it allows us to define the probability density function of the swimmer separations. That is, the probability for a pair to be separated by a certain distance, as shown in Fig. 3d, where the relative energy is $U(\ell )/{k}_{B}T=-\log ({{{{{{{\rm{PDF}}}}}}}}(\ell ))+U/{k}_{B}T$, with *U*/*k*_*B*_*T* an arbitrary reference state. Here, we set this such that the relative energy goes to 0 at infinite separation, where we ignore the periodicity imposed by the post about which the swimmers orbit.

The shape of the resulting effective potentials close to the minimum resembles a harmonic function, as expected, and we fit these with $y=\frac{1}{2}k{(x-{x}_{0})}^{2}+{y}_{0}$ using a least-squares fit. This provides us with the depth of the potential well *y*_0_, the preferred distance *x*_0_, and the interaction strength *k*, see Supplementary Table 1 for values. Our data suggest that the higher the preferred separation is, the weaker the coupling becomes. This could be indicative of differences between the individual participating swimmers, though a simple model, to which we will turn now, suggests another explanation.

### Model for activity-induced interactions

For our modeling, we assume that there is a short-range repulsion, due to the self-propulsion mechanism, and—in view of our recent work^46,47^, as well as other experimental evidence^56^—a long-ranged attraction, due to flow along the substrate. In addition, we assume extremely fast relaxation of the flow and chemical fields with respect to this motion. To lowest order, the short-ranged repulsion is taken to be point-like dipolar in nature, with the sign of a pusher-type swimmer, based on recent experimental results for the flow around isolated chemical swimmers^55^. That is, there is a near-field flow directed outward along the symmetry axis of the swimmer^55^ that scales as ${u}_{{{{{{{{\rm{dip}}}}}}}}}(r,\theta )=\kappa \left(3{\cos }^{2}\theta -1\right)/(2{r}^{4})$. The osmotic flow along the surface is directed inward and scales as *u*_osm_(*r*) = − *λ*/*r*^2^ ^57^, again to lowest order. Here, the factors *κ* and *λ* indicate the respective strength of the outward and inward flows, *r* is the radial distance—the power of the decay accounts for the presence of a no-slip surface above which the swimmer moves—and *θ* is the angle with respect to the swimmer’s orientation.

When the two contributions balance at a finite distance, comoving swimmers can assume a stable separation, see Fig. 3f for a vector plot of the total velocity profile *u*_tot_(*r*, *θ*) = *u*_osm_(*r*) + *u*_dip_(*r*, *θ*) around a single swimmer. The angular dependence shows a lemniscate zero-velocity contour. Clearly, our simple argument would allow for swimmer contact at a finite angle, without introducing further finite-size corrections. This situation can be stabilized by imposing 1D head-to-tail alignment, as induced by the presence of the post in the experiment. Henceforth, we therefore examine only the flow along the *x*-axis. Figure 3g reveals that when aligned head to tail, there is indeed a separation *r*_0_ that is stable, as indicated by the slope at the intercept. For swimmers comoving in the same direction we obtain a simple expression for the separation as a balance between the inward and outward flow strength: ${r}_{0}=\sqrt{\kappa /\lambda }$, see Fig. 3h. Around this point the profile can be recast into an effective quadratic potential, as expected, justifying the fit in Fig. 3d. Note that the swimmers move at a fixed distance in the comoving frame, and that our model does not indicate what the collective speed is.

A more detailed calculation based on Faxén’s first law can additionally reproduce the observed properties of trains of swimmers, especially when the center of the monopolar flow is shifted toward the aft of the swimmer, see the SI and Supplementary Software. The shift modification can be justified by the off-center (hemispherical) production of chemical gradients. Our extended simple model results in closer spacing between leading than trailing swimmers, as our data in Fig. 3d suggests. This model further predicts a collective speedup of the train, though the speed increase is not linear in the number of swimmers, suggesting that additional contributions are needed to capture the experimental observations from two and three microswimmers. Lastly, it should be noted that when one particle is immobilized, our model requires an approaching second particle to have a much smaller separation before repulsion and attraction balance. This is because the repulsion must now also overcome the force imposed by self-propulsion. The particles could even come close to contact, depending on the nature of the electrostatic and steric repulsion. We have not accounted for these effects here as in the swimmer trains the separation is sufficiently large to ignore such short-range interactions. In the case of particles moving toward each other, the effect of the self-propulsion is doubled and particles can approach even closer.

### Formation of chains of microswimmers

**Fig. 4:**
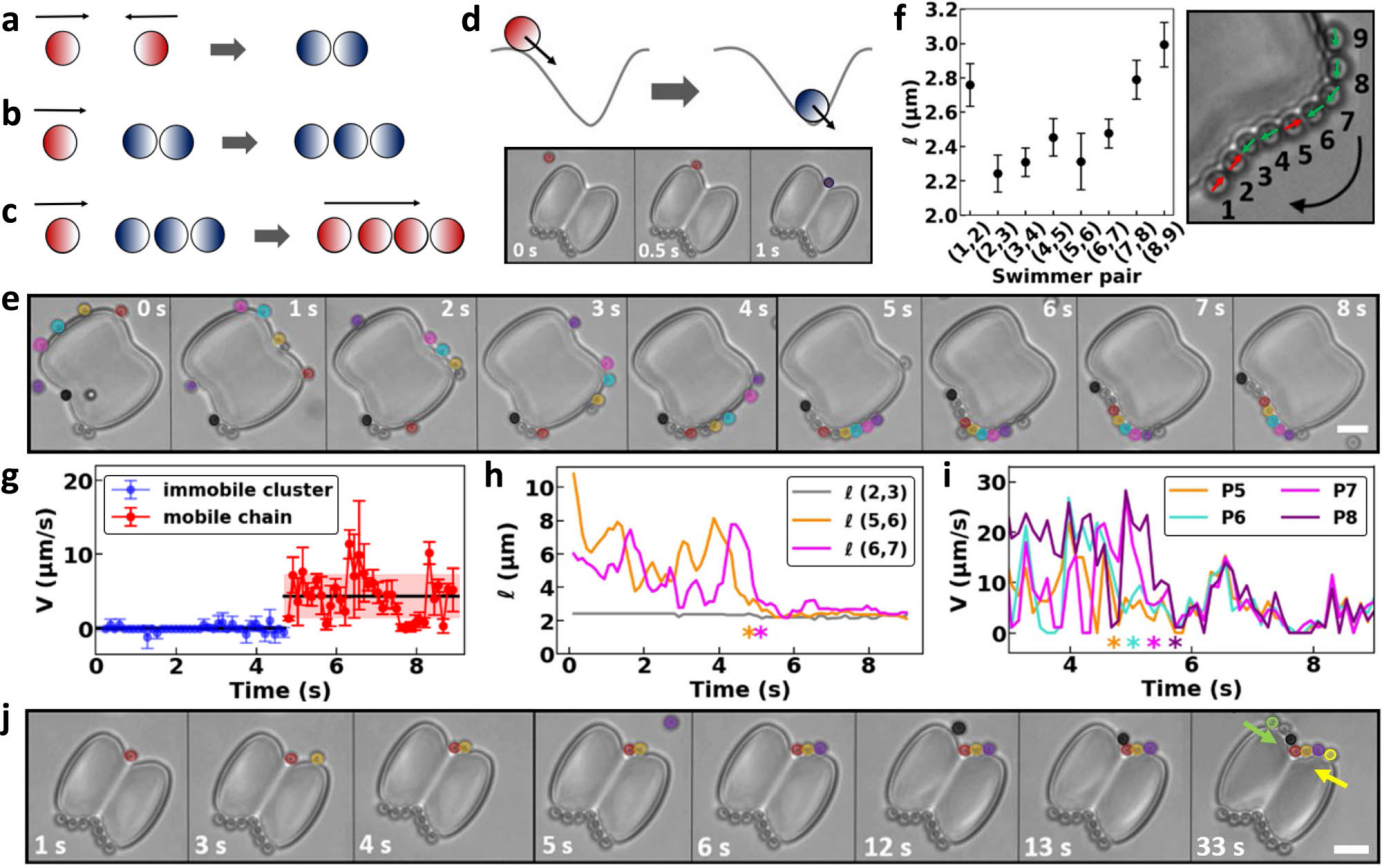
Activity-assembled chains of microswimmers on peanut-shaped posts. **a**–**d** Schematic representations of the conditions determining chain mobility. Red and blue colors indicate mobile and immobilized swimmers, respectively. The darker hemispheres represent the Pt side, which drives motion. **a** Two swimmers with opposing motion direction become immobile. **b** When a third swimmer approaches the cluster, it gets in contact and also becomes stationary. **c** A fourth swimmer, entering from the same side as in **b**, causes the chain to move again in the direction of the majority of swimmers. **d** In highly curved neck regions, swimmers stop temporarily because a reorientation is required, see also the light microscopy image at the bottom. **e** Dynamic chain formation, see also Supplementary Movie 5: initially, self-propelled swimmers headed by the red swimmer move clockwise in a train. After being stopped by an immobile cluster at the lower left edge, the train becomes a compact chain that moves after further addition of swimmers. **f** Center-to-center distance between swimmer pairs varies within a mobile chain. Front of the chain is swimmer 1. Particle numbering, direction of motion of the entire chain (curved arrow), and direction of motion of the individual particles (straight arrows, red is for counterclockwise and green for clockwise direction) are indicated in the bright field image on the right. **g** Speed of the chain at the bottom-left post corner in panel (**e**) as a function of time. While initially being immobile, the chain remobilizes upon addition of swimmers at *t* = 5s. **h**, **i** Particle labeling follows panel (**f**). Asterisks indicate the time at which the individual swimmer (in **h**) or the front swimmer of the pair (in **i**) met the chain. **h** Distances of swimmer pairs that orbit the peanut-shaped posts shown in panel (**e**). The train of particles compacts into a chain upon collision with the immobile cluster on the bottom-left corner; once swimmers are part of the chain, their relative distance remains roughly constant even when the chain gets remobilized. **i** Speed of individual swimmers from panel (**e**) in time. Speeds of swimmers in a train fluctuate, whereas all swimmers move at the same speed in a chain. **j** Posts with a more negative neck curvature enhance the formation and pinning of compact and immobile chains, see also Supplementary Movie 6. This time series is a continuation of the one in panel (**d**).

Our experiments allow us to test these predictions by looking for configurations that disrupt the stable swimmer distance by temporarily or even permanently stopping one or more of the swimmers. One of the blue-colored posts in Fig. 1a shows a pair of swimmers that presumably moved in opposite directions around the post before their encounter immobilized them, see Fig. 4a for a schematic drawing. We also observed immobile clusters consisting of three particles in Fig. 1a. The third swimmer was able to approach the contacting pair of swimmers much closer than the previously observed 3 μm minimum distance in a train, in line with the intuition provided by our minimal model. Intriguingly, the presence of the third swimmer proved insufficient to remobilize the cluster, despite the uneven particle number and hence presumably unbalanced forces, see Fig. 4b.

To gain better control over the location and duration of the stops and to test our hypotheses, we employed microprinted posts with a peanut shape that feature regions of alternating positive and negative curvature. In addition, these larger posts (long axis: 22 μm, short axis: 16.5 μm) allow us to study the interactions and behavior of more than three particles. Although microswimmer speed is independent of the absolute curvature, see Fig. 1c, changes in curvature do affect the motion of the swimmers^16^. Swimmers passing through negative curvature points need time to reorient themselves to be able to move on, see Fig. 4d. The more curved the neck regions of these posts, the longer it takes for the swimmers to escape.

We verified that swimmers with opposing directions of motion hinder each other around our peanut-shaped post, see the bottom edge of the peanut-shaped post in Fig. 4e (*t* = 0 s). Thus, these posts allow for observations that are similar to the ones made for the circular posts, as well as in previous work on channels^15^. Here too, a third swimmer is unable to disturb an immobilized pair configuration and joins the immobile cluster, see the bottom edge of Fig. 4e (*t* = 1 s), where the black-colored swimmer joins the immobile dimer. A fourth swimmer joining the now immobile trimer from the right leads to a balanced situation and an immobile tetramer cluster, see the red-colored swimmer in Fig. 4e at *t* = 2 s and *t* = 3 s. We found that a fourth swimmer is able to remobilize an immobile trimer in our experiments, whenever there are three particles pointing in the same direction, see the sketch in Fig. 4c. We note that swimmers pointing in the direction opposite to the net motion do not need to reorient, they are simply pushed along. We refer to the tetramer (and similar compact configurations like it) as a chain, as the swimmer separation is small. This helps distinguish these clusters from trains, which feature well-separated swimmers.

Generally, we find remobilization of chains whenever the number of swimmers pointing in one direction exceeds the number of swimmers pointing in the other direction by Δ*n* = 2. This is an unexpected result, as naively one would expect a single additional swimmer to be able to push forward an immobilized pair, albeit slowly. We hypothesize that the opposing pair interacts strongly with the surface and two (additional) swimmers having the same direction of travel are minimally required to overcome this adhesion. This information can be used to infer the direction of swimmers throughout the chain, when combined with information on the average separation. For example, swimmers at the ends of long immobile chains always must have directions that point toward its center, while a moving chain requires the trailing swimmer to be oriented in the direction of motion. Conversely, in this manner we can also predict the dynamics of a compact chain upon addition of a swimmer.

The larger size of our peanut-shaped microprinted posts enables the attachment of multiple moving swimmers that can actively interact and dynamically self-assemble and disassemble. This allowed us to see how swimmers who move in trains along the post evolve into compact swimmer chains, see Fig. 4e and Supplementary Movie 5. For example, Fig. 4e (*t* = 0 s) shows that four swimmers who move in the same clockwise direction form a train led by the orange-colored swimmer. Between *t* = 0 s and *t* = 1 s a fifth swimmer, initially swimming on the top of the post, enters the train in between the yellow and orange one. One by one, the swimmers in the train encounter and join the immobilized pair in the bottom-left corner. Due to this stopping point, the swimmers achieve close contact, which causes the train to transition into a chain. Once the number of clockwise-moving swimmers is at least by two greater than the number of counterclockwise moving swimmers in the immobile cluster, the entire chain sets into motion (*t* = 5 s) and moves with an average velocity of (4.4 ± 2.9) μm/s, see Fig. 4g. Since the majority of swimmers are moving clockwise, the clockwise direction is imposed on the chain as a whole.

### Microswimmer distances and speed in chains

After the chain is formed and remobilized, the distance between a swimmer and its neighbors depends on its position in the chain, see Fig. 4f. Swimmers at the chain ends are further apart from their neighbors than the ones in the middle, which nearly touch. Swimmers at both ends are positioned at a center-to-center distance of ≈(2.9 ± 0.2) μm from their neighbors, unlike the swimmers within the chain that move at distances of ≈(2.4 ± 0.3) μm. Since our swimmers have a diameter of (2.00 ± 0.05) μm, this implies that particles in the center of the chain are almost touching. The separation could be due to the pusher-type flow or even involve short-ranged interactions, such as electrostatic repulsion. Note that the distance of the swimmer pairs at the chain ends coincides with the minimum distance found for the swimmer pairs in the circular posts in Fig. 3b. This observation further corroborates our hypothesis of a long-range attraction being present between the swimmers, which is balanced by a short-range repulsion. Because the attraction spans more than a single swimmer, the swimmers in the middle are more compacted than those at the end. In addition, the direction of the swimmer with respect to the direction of motion of the chain impacts their distance, as can be seen for swimmer 5 which features a smaller distance with swimmer 6, Fig. 4f.

A closer examination of individual swimmers that comove in a train along the peanut-shaped path confirms that the distance between swimmers fluctuates around a preferred distance, see Fig. 4h, similar to our findings along spherical posts (Figs. 2 and 3). That is, in the absence of a disturbance such as encountering a particle that moves in the opposite direction or a stationary cluster, particles comoving in a train along closed paths keep at preferred distances. The separation in the orange-turquoise and turquoise-magenta swimmer pairs shown in Fig. 4e fluctuates around (6.2 ± 1.5) μm and (5.0 ± 1.4) μm, see the respective orange and magenta lines in Fig. 3h. After the leading swimmer of each pair is incorporated into the chain (indicated by the asterisks of the respective color in Fig. 4h), swimmers assume a much closer spacing with comparably small fluctuations around their mean. In transitioning to the chain state, individual swimmer speeds adjust to the collective speed of the chain, see Fig. 4i and Supplementary Fig. S3 for a full time series.

Note that the outer swimmers of the chain (swimmers 7 and 8 in Fig. 4i) initially move faster than the swimmers leading the chain (swimmers 5 and 6 in the same figure). This is indicative of a collective speedup, similar to the one we observed previously for swimmers orbiting small circular posts. However, the strongly varying curvature along the peanut-shaped path prevents us from pinpointing the dynamics. This effect is likely also the reason behind the strong fluctuations in the speeds shown in Supplementary Fig. S3.

The peanut-shaped posts also allow us to exploit the effect of the local variation in curvature by printing peanut-shaped posts with stronger cusps, see Fig. 4j. In this case, it is evident that the more highly curved necks act as permanent stopping points, see Supplementary Movie 6. This is reminiscent of the immobilization of swimmers in wedge-like geometries^58–61^. The higher curvature does not prevent the formation of long chains, although these assemble and remain pinned at the neck, see Fig. 4d, j.

### Dynamics of activity-assembled chains

**Fig. 5:**
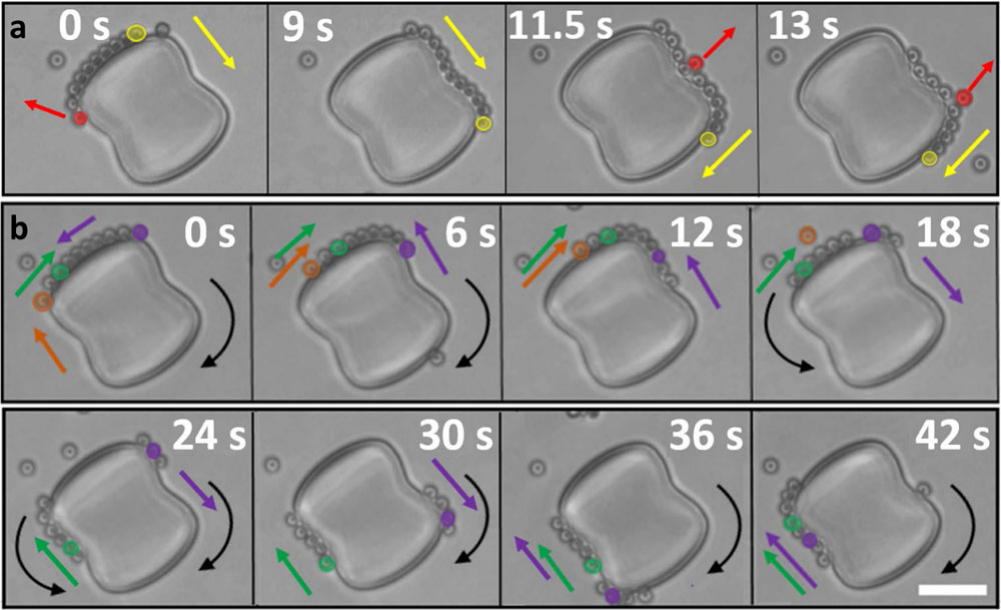
Dynamics of activity-assembled chains: effect of post curvature on chain mobility. **a** A swimmer leaves from the end of the self-propelled compact chain when it reaches the rounded post edge (*t* = 0 s). Swimmers from the middle of the chain escape on the neck or the rounded post edge (*t* = 11.5 and 13 s). Red particles and arrows mark swimmers escaping the chain. See Supplementary Movie 7. **b** Local curvature variation facilitates pinning and chain breakup. The same colors correspond to the same swimmer within each time series, except the red color in **a**. Colored arrows denote the direction of motion of individual swimmers with the corresponding color. Curved black arrows indicate the motion of the chain(s) as a whole, absence of curved arrow denotes that the chain momentarily remained stationary. See also Supplementary Movie 8.

Besides an activity-induced self-assembly into compact chains, chains may also reorganize in time, see Supplementary Movie 7. In Fig. 5a we follow a clockwise self-propelling chain consisting of ten swimmers. While orbiting, swimmers may leave the chain in the following ways: (1) swimmers at the chain end may leave when they reach locations of comparatively high positive or negative curvature, in line with earlier findings for individual swimmers^15,16^. These departures are likely facilitated by their larger distances to their neighbor, see Fig. 4f. This is the case for the red swimmer at *t* = 0 s in Fig. 5a, which leaves the chain when it reaches the rounded peanut edge (top left). (2) Likewise, swimmers from the middle of a chain may exit when they pass through locations where curvature varies. This scenario is visible both at *t* = 11.5 s and *t* = 13.0 s in Fig. 5a, where a mid-chain swimmer highlighted in red escapes while passing through the negatively curved neck and positively curved corner, respectively. We speculate that this is enhanced for swimmers with directionality that opposes the direction of motion of the chain. In both cases, the chain slowed down before the escape, confirming our expectation that curvature variations can induce (local) slow-downs.

In addition to facilitating swimmer escape, this local slow-down can also enhance chain breakup and motion reversal, see Fig. 5b and Supplementary Movie 8 for an example. A clockwise-moving chain becomes pinned at the rounded edge (*t* = 6 s), where some particles leave and others join the chain. This leads to a breakup into two smaller chains, that remobilize and move in opposite directions (*t* = 18 s). Once more, at a point where the local curvature changes its sign, but this time at the neck, one chain gets pinned. The other chain joins and they continue together in the clockwise direction, thereby inducing a second reversal of motion for the chain containing the green particle.

## Discussion

In summary, we have shown that catalytically self-propelled microswimmers exhibit a number of striking collective effects in 1D environments. When moving in the same direction microswimmers cooperate. That is, they move at a greater speed the more particles comove in a train-like structure. Inside the trains, swimmers assume a preferred, significant separation. The activity-induced interaction that induces these effects can be described by an effective interaction potential of the order of few *k*_*B*_*T*. We have provided a theoretical understanding of the train formation using a simple model. In this model, the spacing is caused by a balance between outward pusher flows emanating from the swimmers and inward osmotic flows along the wall, which are caused by the chemical gradients formed in the swimming process.

Stopping a train can cause the comoving swimmers to overcome their preferred spacing, thereby leading to a much more compact structure we refer to as a chain. These active chains show very rich dynamics, including activity-induced self-assembly, compaction, disassembly, breakup, and reformation. A simple rule appears to distinguish immobile from mobile chains: mobility is achieved if the difference in the number of opposing swimmers is greater or equal to two. Once in close proximity, there is still a balance between repulsion and attraction.

Lastly, we found that variation in the sign of local curvature leads to changes in the speed of the swimmers comprising a chain or train, which are absent when the curvature is constant. This dependence of the chain dynamics on the curvature variation can be exploited to facilitate chain compaction and breakup, as well as to immobilize the chains. Using peanut-shaped posts we have shown that this can be achieved at well-defined locations. These aspects offer an exciting route toward more complex forms of manipulating 1D swimmer self-assemblies, including the deliberate formation of defects, and hence answering fundamental questions on their activity-induced phase behavior.

Many other synthetic swimmer systems feature both swimming-induced flows and flows along the surface resulting from gradients generated by their propulsion mechanism, e.g., temperature gradients, osmosis and micelle formation. We expect our findings on the activity-induced 1D swimmer assemblies of trains and chains and the ways in which they can be manipulated to apply to these systems as well, albeit with spacings and speedups unique to the specific propulsion mechanism. Our insights into interactions and collective behavior of synthetic microswimmers could be pivotal for applications that require increased swimming efficiency or directionality across different environments.

## Methods

### Particles

Spherical latex particles based on polystyrene (2% cross-linked) with diameter (2.00 ± 0.05) μm, i.e., size polydispersity 2.5%, were purchased from Sigma Aldrich. Pt-half-coated particles were produced through physical vapor deposition^46,47^ as follows: particles were spin coated from ethanol on glass slides at sub-monolayer concentrations and subsequently sputter coated from above with a (4.7 ± 0.2) nm Pt layer (Pt/Pd 80/20, MicrotoNano70-PPS708) using a standard sputter coating system (Cressington 208HR High Resolution Sputter Coater). The particles were redispersed in water by sonication and were subsequently washed and stored in water.

### 3D printed structures

Microstructures were produced with the commercially available microprinter Photonic Professional GT of Nanoscribe which uses two-photon lithography. The microprinter was equipped with a 63X oil-immersion objective (Zeiss, NA = 1.48) and used to print the 3D structures in oil mode. Microstructure designs were performed in Autodesk Inventor and processed with Describe. The microstructures were printed onto glass coverslips, pre-cleaned with isopropanol, using the commercial photoresist IP-L as a pre-polymer. After printing, the structures were developed by submersion in propylene glycol methylether acrylate for 15 min, followed by gently dipping into isopropanol three times to remove the unpolymerized photoresist. The structures were subsequently dried with gentle air flow. All procedure was done under yellow light.

### Imaging

Pt-half-coated particles were dispersed in a 10% aqueous H_2_O_2_ solution. Their motion was recorded above the planar walls with a ELWD 60x objective (S Plan Fluor, NA 0.7, zoomed at ×1.5, i.e., 0.1 μm/px) mounted on an inverted Nikon Eclipse Ti microscope at a frame rate of 5 and 9 fps along the circular and peanut-shaped posts, respectively, within the first hour after sample preparation.

### Analysis

Particle positions above the planar wall and along the circular posts were obtained using the Python tracking algorithm Trackpy^51^. The speed of all particles was determined using the time derivatives of spatial displacements at consecutive frames, see inset of Fig. 1d for the speed distribution of a single particle measured in orbit for ≈4 min (>1200 frames). For swimmers on circular posts, the (arc) displacement was obtained according to Fig. 3a. On the peanut-shaped posts, distances were obtained using the NIS-Elements Advance Research software package by Nikon. Particle positions and swimmer velocities along the peanut-shaped posts were obtained using ImageJ. The Pearson correlation coefficient is a normalized version of the covariance of two datasets that measures the linear relationship between variables; it ranges between –1 and 1, where –1 indicates negative and +1 positive correlation, and 0 indicates no linear correlation. The Pearson coefficients were computed using the Python scipy.stats module. All errorbars denote standard deviations.

## Supplementary information

Supplementary Information

Description of SI files

Supplementary Movie 1

Supplementary Movie 2

Supplementary Movie 3

Supplementary Movie 4

Supplementary Movie 5

Supplementary Movie 6

Supplementary Movie 7

Supplementary Movie 8

Supplementary software

## Data Availability

All data needed to evaluate the conclusions in the paper are present in the paper and Supplementary Information. Additional data related to this paper may be requested from the corresponding author.

## Code availability

The code containing calculations used for understanding the collective effects of comoving microswimmers (shown in Fig. 3f–h) is available as Supplementary Software.
