# Supplementary material for: Activity-induced interactions and cooperation of artificial microswimmers in one-dimensional environments
Source: Nat Commun. 2022 Apr 1;13:1772. doi: 10.1038/s41467-022-29430-1 (PMC8976030; doi:10.1038/s41467-022-29430-1)
Supplement: Supplementary file 1 — Supplementary Information [file 41467_2022_29430_MOESM1_ESM.pdf]

Supplementary Information for: Activity-induced microswimmer interactions and cooperation in one-dimensional environments

Stefania Ketzetzi, Melissa Rinaldin, Pim Dröge, Joost de Graaf,

Daniela J. Kraft

Comoving swimmers speeds

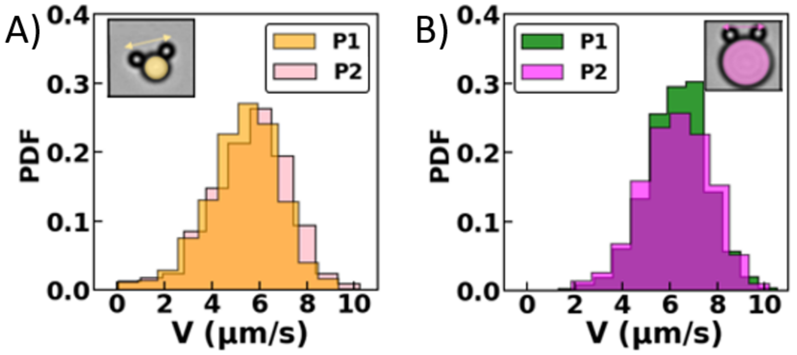

Supplementary Figure 1: Probability density function of the speeds of two swimmers moving along a **A)** 4 µm and **B)** 8 µm post. The front and back swimmer are labeled P1 and P2, respectively. Swimmers orbiting the same post have almost the same speed distribution, independent of swimmer number and post size, see also Figure 2E for three swimmers on a 4 µm post.

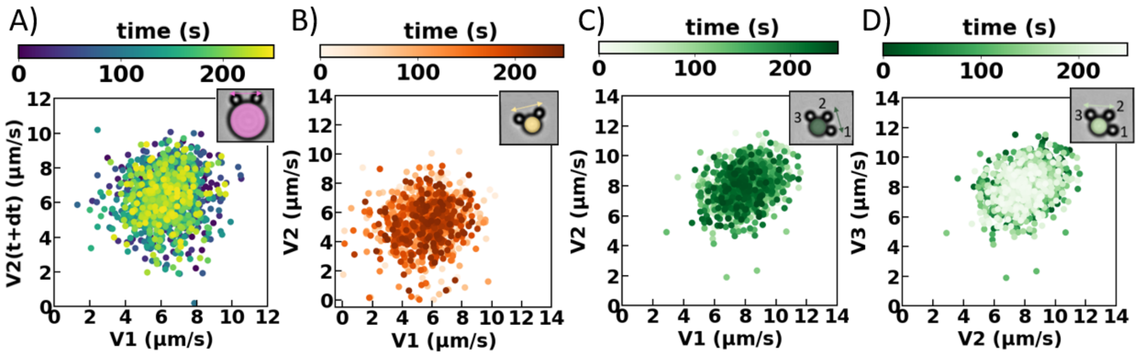

Supplementary Figure 2: **A)** We check for time-delayed correlations by considering the correlation between $V_1(t)$ and $V_2(t+\tau)$, with τ the time between two frames, for the same swimmer pair as in Figure 3E. The Pearson correlation coefficient is 0.1, signifying no linear correlation. Additional scatter plots of the speeds of **B)** two swimmers comoving along a 4 µm post, **C)** the front "1" and middle "2" swimmers and **D)** the middle "2" and back "3" swimmers from a three-swimmer system along a 4 µm post, none of which show a correlation.

Post diam. (µm)	k (k_BT/µm^2)	x_0 (µm)	y_0 (k_BT)	Curve color
4	0.8 ± 0.1	5.05 ± 0.06	-4.4 ± 0.1	orange
8	4.7 ± 0.7	4.15 ± 0.04	-5.9 ± 0.2	magenta
4	8.3 ± 0.5	3.80 ± 0.02	-6.1 ± 0.1	dark green
4	3.9 ± 0.3	4.20 ± 0.02	-4.60 ± 0.04	light green

Supplementary Table 1: Parameters obtained from fitting the data of Figure 3D with $y = \frac{1}{2}k(x - x_0)^2 + y_0$, with k the interaction strength, x_0 the preferred distance and y_0 the potential well depth. From left to right, the columns provide: post diameter, k, x_0, y_0, and the color used for each curve in Figure 3D.

Motion in trains and subsequent chain formation along 1-dimensional paths of nonuniform curvature

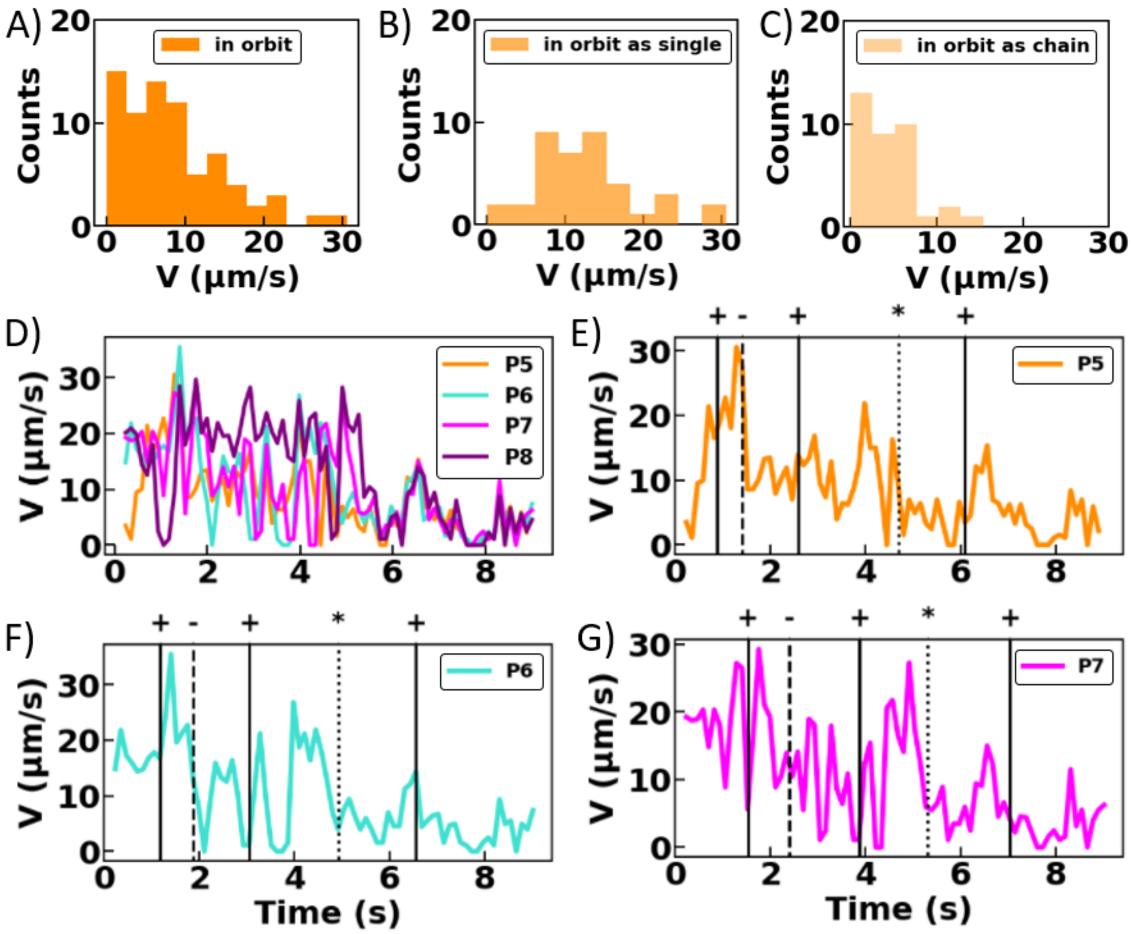

Supplementary Figure 3: Distribution of velocities of a single microswimmer orbiting the peanut-shaped post of Figure 4E: **A)** Full velocity distribution in orbit combining the data of panels (B,C), **B)** Distribution of the velocity of an individual microswimmer that moves in a train before joining a mobile chain, **C)** Distribution of the velocity of the same microswimmer after it becomes a part of the self-propelling chain. **D)** The full time dependence of the velocity for the same swimmers as in Figures 4E and 4I. Initially all swimmers move collectively in a train, with uncorrelated fluctuations in velocity, and thereafter one-by-one compactify to a chain wherein they move as one, see also Figure 4I. Once they are part of the chain, all swimmers move at the same speed. **E-G)** The velocity of the same microswimmers as in panel (D) in time. These graphs indicate when each swimmer passes through consecutive points of varying curvature as it moves along the peanut-shaped path: solid lines and "+" symbols indicate that the swimmer has reached a post edge corresponding to points of positive curvature while dashed lines and "-" symbols indicate that the swimmer has reached the neck of the peanut-shaped path where the path curvature becomes negative. Dotted lines and "$*$" symbols indicate the point in time when the swimmer becomes part of the mobile chain.

Minimal model for comoving swimmers' distances

Here, we provide an in-depth discussion regarding the origin of the activity-induced interaction, see also Figure 3. Referencing the recent work on two interacting chemical swimmers in bulk [1, 2] that generally there is repulsion between two identical comoving swimmers in bulk. A more detailed analysis of a wide range of couplings [3] reveals that a pure bulk hydrodynamic and chemotactic gap stabilization is possible for specific choices of the surface mobilities. However, in view of our recent work [4, 5], we hypothesize that instead there is a short-ranged repulsion due to the swimming mechanism itself and a long-ranged attraction due to osmotic flows along the substrate. The balance between these two flows then leads to the observed stable separation between swimmers. The attraction must be the longer ranged effect to be commensurate with the particles assembling into co-moving objects, see also discussion on chain formation. The repulsion can be related to the pusher-type nature of the self-propulsion, which induces outward directed fluid flow along the swimmer symmetry axis. When the two contributions balance at a finite distance, comoving swimmers can assume a stable separation. To show that this argument has merit, we formulated a minimal model that exhibits such a balance point.

An inward directed flow due to osmosis along the substrate [6] scales as $u_{\text{osm}}(r) = -\lambda/r^2$, while a pusher-type swimmer has an outward directed flow [7] that scales as $u_{\text{dip}}(r,\theta) = \kappa\left(3\cos^2\theta - 1\right)/(2r^4)$ (the bulk r^2 decay becomes r^4 due to proximity to a no-slip wall). The factors λ and κ indicate the respective strength of the inward and outward flow. Note that we only take the leading-order contributions for both effects in our proof of concept. Clearly, a more detailed calculation could distinguish between various forms of self-propulsion, and account for near-field effects, but this goes beyond the scope of the current investigation. For an appropriate choice of the prefactors $\lambda = 0.1\kappa$ we obtain the total velocity $u_{\text{tot}}(r,\theta) = u_{\text{osm}}(r) + u_{\text{dip}}(r,\theta)$ profile shown in Figure 3G of the main text, which indeed reveals a favorable separation r_0. Generally, we have that $r_0 = \sqrt{\kappa/\lambda}$ for comoving swimmers that are pointed in the same direction, see Figure 3H. The slope of the velocity profile at the intercept in Figure 3G indicates a stable configuration. Thus, we expect that this profile can be recast into an effective potential, as was done from the distance data in the experiment, see also Figures 3C and 3D. Setting $\theta = 0$ and locally linearizing $u_{\text{tot}}(r - r_0) \approx -Us$ with $U > 0$ the absolute value of the slope and $s = r - r_0$ the perturbation around the favorable separation. In the low-Reynolds number regime, a speed can be converted into a force via the mobility, i.e. $F = -6\pi\eta a U s$ in this case, with η the viscosity and a the sphere radius. The sign indicates the restorative nature of the force, with $U > 0$ as before. If F is a force deriving from an effective potential, then the potential has the shape $3\pi\eta a U s^2$, which is the origin of the harmonic potential inferred from the experiment. When one of the particles is immobilized, the second particle may approach much more closely, possibly even come into contact (depending on the strength of the repulsion versus self-propulsion), and similarly for particles moving toward each other.

Thus far, we have focused on co-moving point particles. If we instead consider finite size particles, we need to account for additional speed-up terms. We can do so by employing Faxén's first law [8], which in the absence of bulk flow can be cast to read:

$$\Delta \boldsymbol{U} = \left(1 + \frac{a^2}{6}\nabla^2\right)\boldsymbol{u}'(\boldsymbol{r}). \tag{1}$$

Here, $\Delta \boldsymbol{U}$ the change in speed of a spherical particle with radius a, as a consequence of the flow $\boldsymbol{u}'$ induced by other swimmers at the center $\boldsymbol{r}$ of the swimmer. The symbol ∇ denotes the gradient and ∇^2 is the vector Laplacian. We can straightforwardly compute the value of the separation for which the particles co-move and this turns out to give a very similar result to our much simpler zero-velocity, point-particle argument, see the Mathematica notebook that is part of the Supplemental material.

The argument for triplet and higher-order terms is similar. The notebook that is a part of this submission provides the relevant expressions for the triplet. Using the Faxén argument, we do not find a collective speedup. However, this may be realized by shifting the origin of the inward directed flow along the substrate to the rear of the particle. A possible justification of this is the Pt-cap being at the rear of the swimmer [9, 10, 11]. In that case, there is also a heterogeneous distribution of particles in a chain, with the leading particles being more closely spaced than the trailing ones, in line with our experimental observation. However, we should emphasize that this is a minimal qualitative capture of the observed behavior, rather than an explanation that accounts for the details of the experiment.

Lastly, turning to the speed-up of multiple particles moving in the same direction: it is important to note that $u_{\mathrm{tot}}(r)$ describes a relative speed, and our argument is robust to a change in the net speed of the pair. Such a collective speedup may be the result of the long-ranged chemical gradients leading to an enhancement of the phoretic driving mechanism, or a collective hydrodynamic effect, such as a drag reduction for the actively-assembled dimer and trimer [12].

References

[1] Sharifi-Mood, N., Mozaffari, A. & Córdova-Figueroa, U. M. Pair interaction of catalytically active colloids: from assembly to escape. *J. Fluid Mech.* **798**, 910 (2016).

[2] Varma, A. & Michelin, S. Modeling chemo-hydrodynamic interactions of phoretic particles: A unified framework. *Phys. Rev. Fluids* **4**, 124204 (2019).

[3] Nasouri, B. & Golestanian, R. Exact axisymmetric interaction of phoretically active janus particles. *J. Fluid Mech.* **905**, A13 (2020).

[4] Ketzetzi, S., de Graaf, J., Doherty, R. P. & Kraft, D. J. Slip length dependent propulsion speed of catalytic colloidal swimmers near walls. *Phys. Rev. Lett.* **124**, 048002 (2020).

[5] Ketzetzi, S., de Graaf, J. & Kraft, D. J. Diffusion-based height analysis reveals robust microswimmer-wall separation. *Phys. Rev. Lett.* **125**, 238001 (2020).

[6] Uspal, W. E., Popescu, M. N., Tasinkevych, M. & Dietrich, S. Shape-dependent guidance of active janus particles by chemically patterned surfaces. *New J. Phys.* **20**, 015013 (2018).

[7] Campbell, A. I., Ebbens, S. J., Illien, P. & Golestanian, R. Experimental observation of flow fields around active janus spheres. *Nat Comm* **10**, 3952 (2019).

[8] Faxén, H. Der widerstand gegen die bewegung einer starren kugel in einer zähen flüssigkeit, die zwischen zwei parallelen ebenen wänden eingeschlossen ist. *Annalen der Physik* (1922).

[9] Ebbens, S. J. & Howse, J. R. Direct observation of the direction of motion for spherical catalytic swimmers. *Langmuir* **27**, 12293–12296 (2011).

[10] Simmchen, J. *et al.* Topographical pathways guide chemical microswimmers. *Nat. Comm.* **7**, 10598 (2016).

[11] Das, S. *et al.* Boundaries can steer active janus spheres. *Nat. Comm.* **6**, 8999 (2015).

[12] Reichert, M. & Stark, H. Circling particles and drafting in optical vortices. *J. Phys.: Condens. Matter* **16**, S4085 (2004).

INFO

Created by J. de Graaf
Date: 26 Sept. 2021
Mathematica version 10.3

SIMPLE VELOCITY ANALYSIS FOR A CO-MOVING DIMER OF SWIMMERS

In this secion we introduce the concept of a chemi-hydrodynamic coupling between swimmers in a train. This consists of simple dipolar flow that accounts for the self-populsion and a monopolar contribution along the surface above which the swimmer moves. The balance between the two resultant flows gives rise to an optimal separation between swimmers in a train.

The monopolar flow along the wall is directed toward the swimmer, hence the minus sign. Here, we assume it is centered on the center-of-mass of the swimmer, which is located in the orgin. The scaling with separation is r^{-2} due to the presence of the wall.The parameter λ provides the effective strength of the interaction. The flow is assumed to be effectively two dimensional, which is a reasonable approximation considering the low-level description used in our model.

In[12]:=
```
uwall = -λ * {xf, yf} / (xf ^ 2 + yf ^ 2) ^ (3 / 2);
uwall // TraditionalForm
```

Out[13]//TraditionalForm=

$$\left\{-\frac{\lambda\,\mathrm{xf}}{\left(\mathrm{xf}^2+\mathrm{yf}^2\right)^{3/2}},-\frac{\lambda\,\mathrm{yf}}{\left(\mathrm{xf}^2+\mathrm{yf}^2\right)^{3/2}}\right\}$$

We capture the hydrodynamic flow field due to a pusher swimmer with a hydrodynamic dipole that decays as r^{-4} in the far field to account for the presence of a wall. The prefactor accounting for the strength is κ.

In[14]:=
```
udip = κ * ((3 * Cos[ArcTan[xf, yf]] ^ 2 - 1) / 2) * {xf, yf} / (xf ^ 2 + yf ^ 2) ^ (5 / 2);
udip // Simplify // TraditionalForm
```

Out[15]//TraditionalForm=

$$\left\{\frac{\kappa\,\mathrm{xf}\left(2\,\mathrm{xf}^2-\mathrm{yf}^2\right)}{2\left(\mathrm{xf}^2+\mathrm{yf}^2\right)^{7/2}},-\frac{\kappa\,\mathrm{yf}\left(\mathrm{yf}^2-2\,\mathrm{xf}^2\right)}{2\left(\mathrm{xf}^2+\mathrm{yf}^2\right)^{7/2}}\right\}$$

From this we compute the net flow in the far-field and plot the result.The blue arrows indicate the in-plane flow lines and the red lemniscate indicates the range where the net flow is zero. Here the ratio $\kappa/\lambda = 10$, which is an arbitrary choice, but the shape is qualitatively similar for all $\lambda,\kappa > 0$.

In[16]:=
```
utot = udip + uwall;

psubs = {λ → 1, κ → 10};
uplot = utot /. psubs;
Uplot = uplot.Normalize[{xf, yf}];
Show[StreamPlot[uplot, {xf, -5, 5}, {yf, -5, 5}, StreamStyle → {Blue, Thick}],
 ContourPlot[Uplot, {xf, -5, 5}, {yf, -5, 5}, Contours → {0.0},
  ContourStyle → {{Thickness[0.01], Red}}, ContourShading → False],
 ImageSize → 500, FrameStyle → Directive[{Black, 20}],
 FrameLabel → {Style["x", Black, 25], Style["y", Black, 25]}]
```

Out[20]=

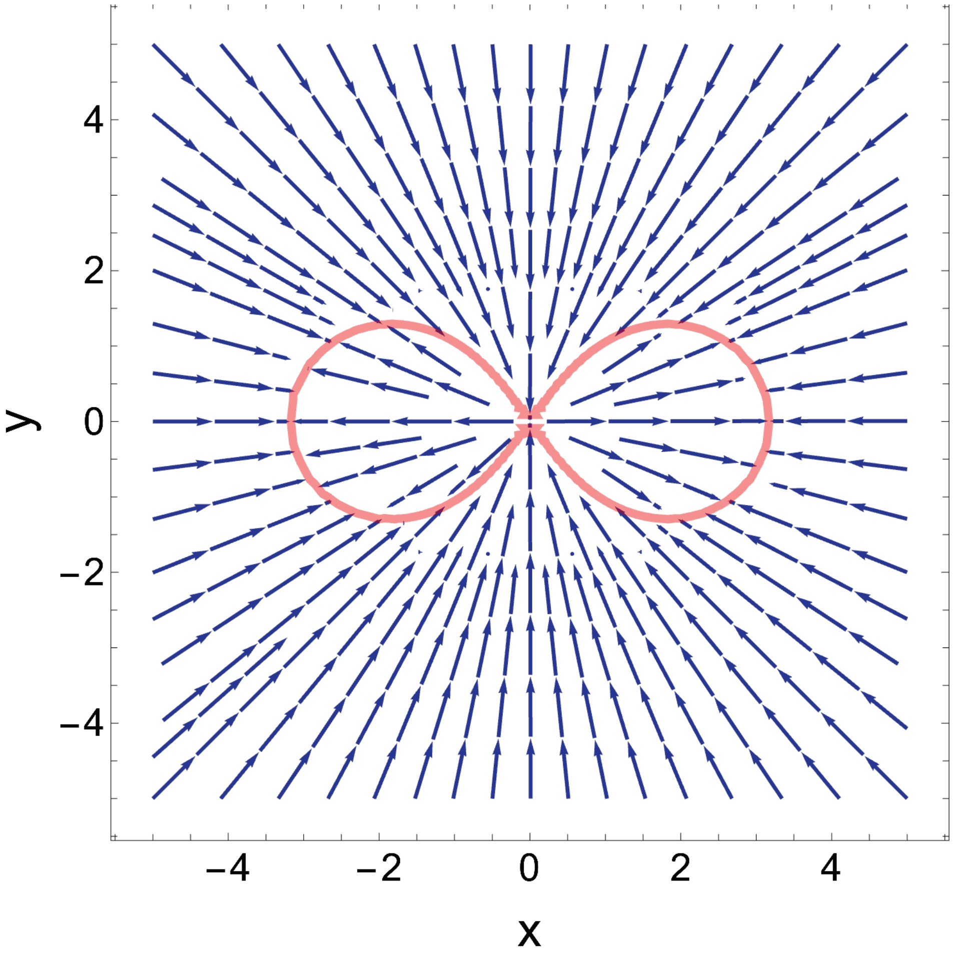

Assuming that the swimmers are pointing head tot tail, we can balance the two contributions of the flow and find the optimal swimmer separation Δx. This gives the expression for the symmetric separation:

In[21]:=
```
ux = utot〚1〛 /. {yf → 0};
xn = Simplify[Solve[ux == 0, xf]〚1, 1, 2〛, Assumptions → {λ > 0, κ > 0}];
xp = Simplify[Solve[ux == 0, xf]〚2, 1, 2〛, Assumptions → {λ > 0, κ > 0}]
FullSimplify[xn == -xp, Assumptions → {λ > 0, κ > 0}]
```

Out[23]= $\sqrt{\frac{\kappa}{\lambda}}$

Out[24]= True

Note that the selection brackets “[[ ]]” above pick the expression for one of the two solutions for the separation. Please verify that the order is correct for your specific version of *Mathematica.*

Visually the flow along the axis is represent by the blue curve in the figure below. The intersection points in red indicate where a second swimmer would be optimally located. Note that this simple calculation does not provide insight in any speed-up or slow-down of the pair, as the only criterion used is whether or not the particles are moving at the same speed.

In[25]:=
```
uline = utot⟦1⟧ /. {yf → 0} /. psubs;
pnts = {{xn, 0}, {xp, 0}} /. psubs;
Show[Plot[uline /. {κ → 10}, {xf, 0.1, 5}, PlotStyle → {Thick, Blue}],
 Plot[uline /. {κ → 10}, {xf, -5, -0.1}, PlotStyle → {Thick, Blue}],
 ListPlot[pnts, PlotStyle → {Thick, Red}], PlotRange → {-0.2, 0.2},
 ImageSize → 750, TicksStyle → Directive[{Black, 20}],
 AxesLabel → {Style["x", Black, 25], Style["u(x)", Black, 25]}]
```

Out[27]=

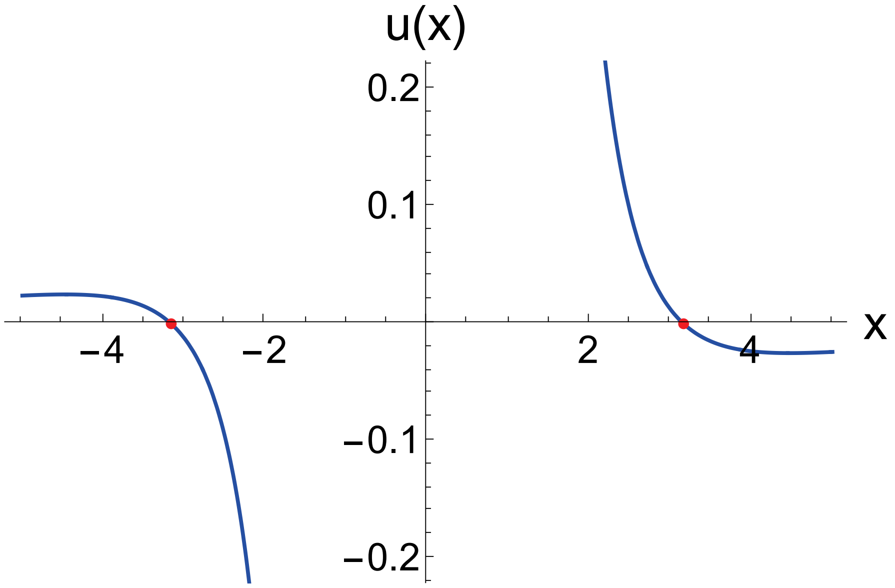

The optimal separation Δx varies with the wall coupling, which an be visualized by varying κ with respect to λ. At this point, our model does not contain a swimmer size, the calculation assumes point-particles. However, we tentatively place the radius of our swimmer at $a = 1/2$, which means that swimmers cannot come closer than their diameter $\sigma = 1$, as indicated by the blue dashed line. The use of a red, and subtle dotted green line shows that the solutions are front-aft symmetric. That is, if a trimer or even a higher-order polymer was formed using this interaction, the distance between all swimmers would be the same.

In[28]:=
```
xplot = xp /. {λ → 1};
yplot = -xn /. {λ → 1};
plotreg = Plot[{1, xplot, yplot}, {κ, 0.5, 10},
  PlotStyle → {{Dashed, Blue, Thick}, {Thick, Red}, {Thick, Green, Dotted}},
  PlotRange → {{0, 10}, All}, ImageSize → 750, TicksStyle → Directive[{Black, 20}],
  AxesLabel → {Style["λ/κ", Black, 25], Style["Δx", Black, 25]},
  AxesOrigin → {0, 0}]
```

Out[30]=

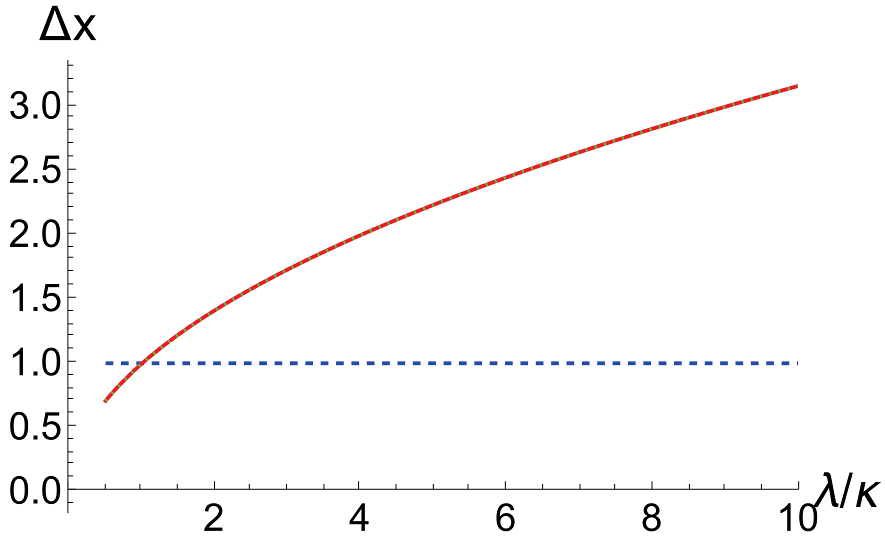

COLLECTIVE EFFECTS AND (POTENTIAL) SPEED-UP BY ACCOUNTING FOR THE SWIMMER SIZE

Clearly the above simple model does not provide a source of speed-up for the train. In fact, speed of the swimmer does not enter the equation, but one could assume that this is proportional to κ. In this section, we introduce the radius a through Faxen’s law. The remainder of the theory is left unmodified, i.e., we still rely on a simple monopolar and dipolar flow that accounts for the presence of a wall.

Faxen’s first law states that the speed change is related to the Laplacian of the velocity field in the center of the sphere (swimmer) being perturbed by an external flow with prefactor $a^2/6$ in addition to the effect of advection acting on a point particle. This is captured by the calculation below. Note that we compute the effect of the front swimmer on the aft swimmer and vice versa, respectively. In each line, the swimmer acting on the other is located in the orgin and the separation is denoted by ‘z’, which is solved for and represents the separation Δx in the graphs below.

In[31]:=
```
uaft = Simplify[utot /. {xf → x - z, yf → y}];
FaxAft = (uaft + (a^2 / 6) * (D[uaft, x, x] + D[uaft, y, y]));
ΔuAft = Simplify[FaxAft⟦1⟧ /. {y → 0, x → 0}, Assumptions → {λ > 0, κ > 0}]
```

Out[33]=

$$\frac{-2 z^2 \kappa + 2 z^4 \lambda + a^2 \left(-4 \kappa + z^2 \lambda\right)}{2 z^5 \sqrt{z^2}}$$

In[34]:=
```
ufro = Simplify[utot /. {xf → x + z, yf → y}];
FaxFro = (ufro + (a^2 / 6) * (D[ufro, x, x] + D[ufro, y, y]));
ΔuFro = Simplify[FaxFro⟦1⟧ /. {y → 0, x → 0}, Assumptions → {λ > 0, κ > 0}]
```

Out[36]=
$$\frac{2 z^2 \kappa - 2 z^4 \lambda + a^2 \left(4 \kappa - z^2 \lambda\right)}{2 z^5 \sqrt{z^2}}$$

In order to be a stable pair, the front and aft swimmer must be equally sped up or slowed down.

In[37]:=
```
sols = Solve[Simplify[(ΔuAft == ΔuFro)], z]
```

Out[37]=
$$\left\{\left\{z \to -\frac{1}{2}\sqrt{-a^2 + \frac{2\kappa}{\lambda} - \frac{\sqrt{4\kappa^2 + 28 a^2 \kappa \lambda + a^4 \lambda^2}}{\lambda}}\right\}, \left\{z \to \frac{1}{2}\sqrt{-a^2 + \frac{2\kappa}{\lambda} - \frac{\sqrt{4\kappa^2 + 28 a^2 \kappa \lambda + a^4 \lambda^2}}{\lambda}}\right\}, \left\{z \to -\frac{1}{2}\sqrt{-a^2 + \frac{2\kappa}{\lambda} + \frac{\sqrt{4\kappa^2 + 28 a^2 \kappa \lambda + a^4 \lambda^2}}{\lambda}}\right\}, \left\{z \to \frac{1}{2}\sqrt{-a^2 + \frac{2\kappa}{\lambda} + \frac{\sqrt{4\kappa^2 + 28 a^2 \kappa \lambda + a^4 \lambda^2}}{\lambda}}\right\}\right\}$$

Only the real-valued positive solution for the resulting separation of the particles is reasonable. This gives a value of a bit over 3x the diameter when $\kappa = 10\lambda$.

In[38]:=
```
solz = sols⟦4⟧
solz /. {a → 1 / 2} /. psubs // N
```

Out[38]=
$$\left\{z \to \frac{1}{2}\sqrt{-a^2 + \frac{2\kappa}{\lambda} + \frac{\sqrt{4\kappa^2 + 28 a^2 \kappa \lambda + a^4 \lambda^2}}{\lambda}}\right\}$$

Out[39]= {z → 3.21834}

The collective speed-up remains zero, despite the addition of an effective size.

In[40]:=
```
Δu = Simplify[FaxFro⟦1⟧ /. {x → 0, y → 0} /. solz, Assumptions → {a > 0, λ > 0, κ > 0}]
```

Out[40]= 0

Clearly, the dependence of the separation Δx on κ, λ, and a is more complicated than before. Here we visualize the new trend (red dashed line) and show that it is quite similar to our previous result.

In[41]:=
```
xplot = solz[[1, 2]] /. {a → 1 / 2, λ → 1};
plotfax = Plot[{1, xplot}, {κ, 0.5, 10},
   PlotStyle → {{Dashed, Blue, Thick}, {Thick, Red, Dotted}},
   PlotRange → {{0, 10}, All}, ImageSize → 750,
   TicksStyle → Directive[{Black, 20}], AxesLabel →
    {Style["λ/κ", Black, 25], Style["Δx/σ", Black, 25]}, AxesOrigin → {0, 0}];
Show[plotreg, plotfax]
```

Out[43]=

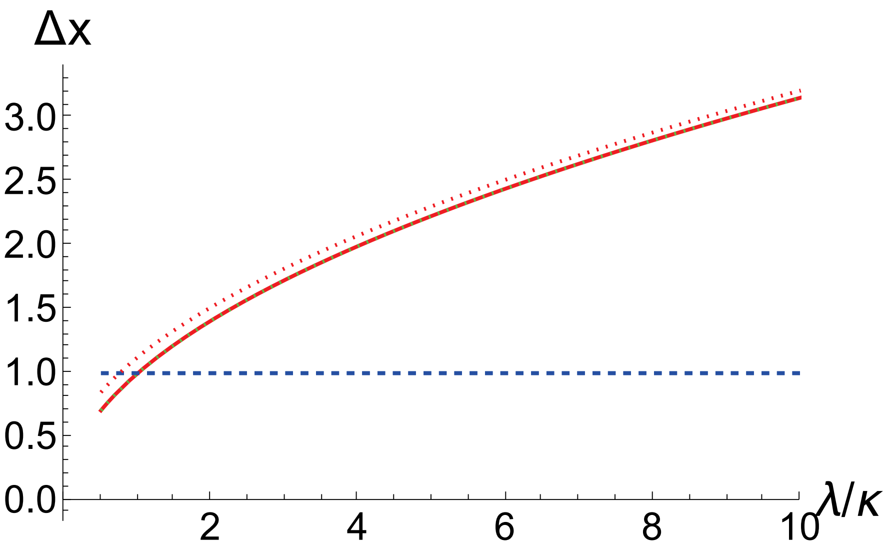

COMPUTING THE PROPERTIES OF A SWIMMER TRIMER

Repeating the above calculation for three swimmers, it is clear that we need to determine the effect of trailing pair on the leading swimmer, the front and aft swimmer on the middle one, and the leading pair on the rear swimmer, respectively.

In[44]:=
```
ufro =
  Simplify[utot /. {xf → x + r, yf → y}] + Simplify[utot /. {xf → x + (r + q), yf → y}];
FaxFro = (ufro + (a^2 / 6) * (D[ufro, x, x] + D[ufro, y, y]));
ΔuFro = Simplify[FaxFro[[1]] /. {y → 0, x → 0},
  Assumptions → {λ > 0, κ > 0, a > 0, q > 0, r > 0}]
```

Out[46]=

$$\frac{\kappa}{r^4} - \frac{\lambda}{r^2} - \frac{2\,q\,r\,\lambda}{(q+r)^4} - \frac{r^2\,\lambda}{(q+r)^4} + \frac{\kappa - q^2\,\lambda}{(q+r)^4} - \frac{a^2\left(-4\,r^6\,\kappa - 4\,(q+r)^6\,\kappa + q^2\,r^6\,\lambda + 2\,q\,r^7\,\lambda + r^8\,\lambda + r^2\,(q+r)^6\,\lambda\right)}{2\,r^6\,(q+r)^6}$$

In[47]:=
```
umid = Simplify[utot /. {xf → x - r, yf → y}] + Simplify[utot /. {xf → x + q, yf → y}];
FaxMid = (umid + (a^2 / 6) * (D[umid, x, x] + D[umid, y, y]));
ΔuMid = Simplify[FaxMid[[1]] /. {y → 0, x → 0},
  Assumptions → {λ > 0, κ > 0, a > 0, q > 0, r > 0}]
```

Out[49]=

$$\frac{\kappa}{q^4} - \frac{\kappa}{r^4} - \frac{\lambda}{q^2} + \frac{\lambda}{r^2} + \frac{1}{2}\,a^2\left(\frac{4\,\kappa}{q^6} - \frac{4\,\kappa}{r^6} - \frac{\lambda}{q^4} + \frac{\lambda}{r^4}\right)$$

```
In[50]:= uaft =
    Simplify[utot /. {xf → x - q, yf → y}] + Simplify[utot /. {xf → x - (q + r), yf → y}];
  FaxAft = (uaft + (a^2 / 6) * (D[uaft, x, x] + D[uaft, y, y]));
  ΔuAft = Simplify[FaxAft⟦1⟧ /. {y → 0, x → 0},
    Assumptions → {λ > 0, κ > 0, a > 0, q > 0, r > 0}]
```

Out[52]=

$$-\frac{\kappa}{q^4}+\frac{\lambda}{q^2}+\frac{q^2\lambda}{(q+r)^4}+\frac{2qr\lambda}{(q+r)^4}+\frac{-\kappa+r^2\lambda}{(q+r)^4}+\frac{1}{2}a^2\left(-\frac{4\kappa}{q^6}+\frac{\lambda}{q^4}+\frac{q^2\lambda}{(q+r)^6}+\frac{2qr\lambda}{(q+r)^6}+\frac{-4\kappa+r^2\lambda}{(q+r)^6}\right)$$

The expressions become relatively complex, hence we solve these numerically for a set of 'standard' parameters. The graph below shows that there is a unique solution satisfying both pairs of conditions, located at the intersection point of the two curves.

In[53]:=
```
subs = Join[{a → 1 / 2}, psubs];
pl0 = Simplify[(ΔuFro - ΔuMid) /. subs];
pl1 = Simplify[(ΔuAft - ΔuMid) /. subs];
Show[ContourPlot[pl0, {q, 1, 5}, {r, 1, 5}, Contours → {0},
   ContourStyle → {{Blue, Thick}}, ContourShading → None],
ContourPlot[pl1, {q, 1, 5}, {r, 1, 5}, Contours → {0},
   ContourStyle → {{Red, Thick}}, ContourShading → None],
 ImageSize → 500, FrameStyle → Directive[{Black, 20}],
 FrameLabel → {Style["Δx12", Black, 25], Style["Δx23", Black, 25]}]
```

Out[56]=

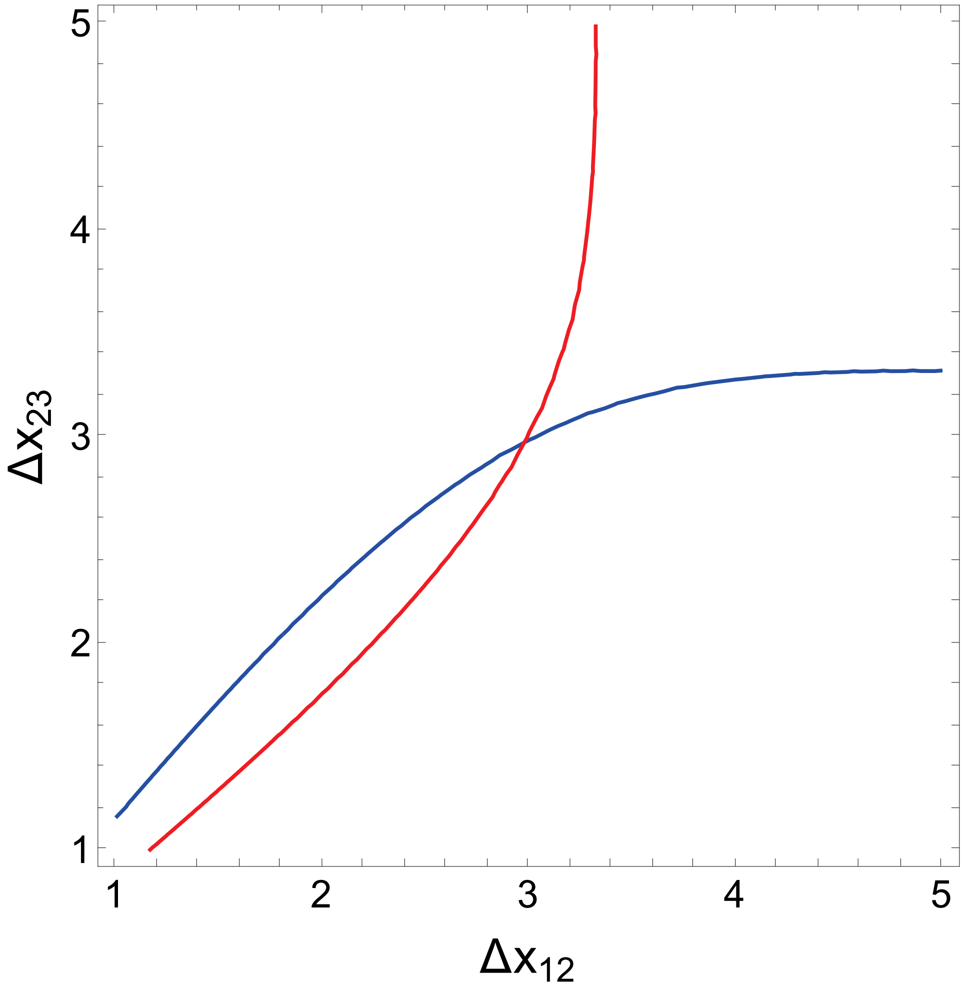

In[57]:=
```
set = ({ΔuFro == ΔuMid, ΔuMid == ΔuAft} /. subs);
solsel = FindRoot[set, {{q, 2, 1, 5}, {r, 2, 1, 5}}]
```

Out[58]= {q → 2.97534, r → 2.97534}

Contrasting with the solution for the dimer, the spacing has reduced somewhat. The new separation is about 92% of that of the dimer. This reduction is expected on the basis of the attractive, monopolar flow having the longest range.

In[59]:=
```
solsel[[1, 2]] / N[solz /. subs][[1, 2]]
```

Out[59]= 0.924495

INTRODUCING A FRONT-AFT ASYMMETRY IN A TRAIN OF SWIMMERS

The following calculation shows that it is possible to introduce a difference in separation and a collective speedup by shifting the center monopolar flow field slightly away from the center. Here we choose to shift the origin of this field toward the aft of the swimmer, where the platinum cap is located. This choice is reasonable, as the platinum cap can be seen as a source of a solute field that is off center. The dipolar term is left unaffected. In the calculation below the parameter ‘s’ is the fraction of the ratius a that the center of monopolar flow field is shifted with respect to the origin.

In[60]:=
```
uwall = -λ * {(xf - s * a), yf} / ((xf - s * a)^2 + yf^2)^(3 / 2);
uwall // TraditionalForm
utot = udip + uwall;
```

Out[61]//TraditionalForm=

$$\left\{-\frac{\lambda\,(\mathrm{xf}-a\,s)}{\left((\mathrm{xf}-a\,s)^2+\mathrm{yf}^2\right)^{3/2}},\,-\frac{\lambda\,\mathrm{yf}}{\left((\mathrm{xf}-a\,s)^2+\mathrm{yf}^2\right)^{3/2}}\right\}$$

The idea of the shift is visualized as follows. The platinum cap is indicated in gray, the inert cap in white, the direction of motion using a red arrow, and an imagined solute field using a green distribution.

In[63]:=
```
nsub = {a → 1 / 2, κ → 5, λ → 1, s → 0.7};
Show[DensityPlot[(Exp[-((xf - s * a) ^ 2 + yf ^ 2)] /. nsub), {xf, -2, 2}, {yf, -2, 2},
   ColorFunction → Function[{z}, Lighter[Green, 1 - z ^ 2]]], Graphics[
   {EdgeForm[{Thick, Black}], White, Disk[{0, 0}, 1 / 2, {Pi / 2, 3 * Pi / 2}]}],
  Graphics[{EdgeForm[{Thick, Gray}], Gray, Disk[{0, 0}, 1 / 2, {-Pi / 2, Pi / 2}]}],
  Graphics[
   {Red, Thickness[0.005], Arrowheads[.05], Arrow[{{1 / 4, 0}, {-1 / 4, 0}}]}],
  ImageSize → 500, FrameStyle → Directive[{Black, 20}],
  FrameLabel → {Style["x", Black, 25], Style["y", Black, 25]}, AxesOrigin → {0, 0}]
```

Out[64]=

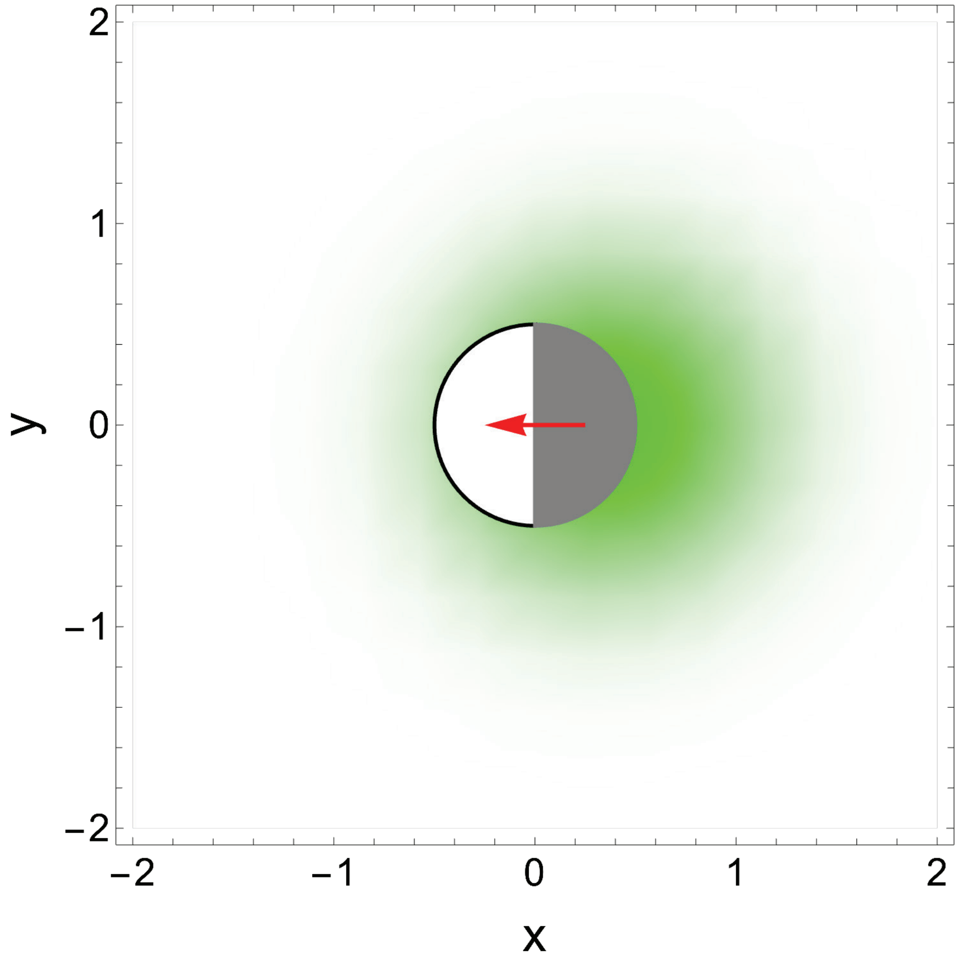

Plotting the flow field, we now find an asymmetric lemniscate indicating the range where the net flow is zero. Here the ratio $\kappa/\lambda = 5$, $a = 1/2$, and the shift is 0.7. The ‘finite size’ of the swimmer is indicated using a dashed, black circle.

In[65]:=
```
uplot = utot /. nsub;
Uplot = uplot.Normalize[{xf, yf}];
Show[StreamPlot[uplot, {xf, -3, 3}, {yf, -3, 3}, StreamStyle → {Blue, Thick}],
 ContourPlot[Uplot, {xf, -3, 3}, {yf, -3, 3}, Contours → {0.0},
  ContourStyle → {{Thickness[0.01], Red}}, ContourShading → False],
 Graphics[{EdgeForm[{Dashed, Thick, Black}], Opacity[0], Disk[{0, 0}, 1 / 2]}],
 ImageSize → 500, FrameStyle → Directive[{Black, 20}],
 FrameLabel → {Style["x", Black, 25], Style["y", Black, 25]}]
```

Out[67]=

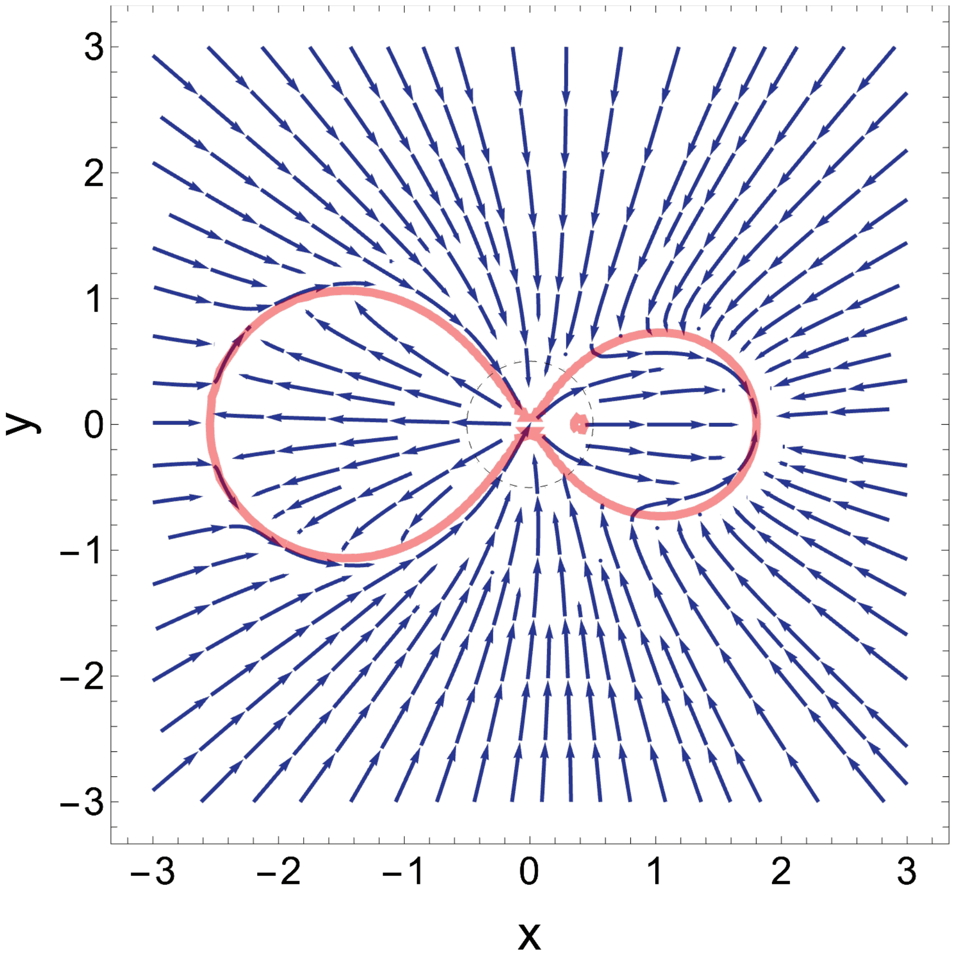

This also leads to an asymmetric arrangement of stable points, not accounting for the effect of size.

In[68]:=
```
uline = utot⟦1⟧ /. {yf → 0} /. nsub;
ux = utot⟦1⟧ /. {yf → 0} /. nsub;
psol = Select[Select[Solve[ux == 0, xf], #⟦1, 2⟧ ∈ Reals &], Abs[#⟦1, 2⟧] > 1 &];
pnts = {{psol⟦1, 1, 2⟧, 0}, {psol⟦2, 1, 2⟧, 0}};
Show[Plot[uline, {xf, -3, -0.1}, PlotStyle → {Thick, Blue}],
 Plot[uline, {xf, 1, 3}, PlotStyle → {Thick, Blue}],
 ListPlot[pnts, PlotStyle → {Thick, Red}], PlotRange → {-0.2, 0.2},
 ImageSize → 750, TicksStyle → Directive[{Black, 20}], AxesLabel →
  {Style["x", Black, 25], Style["u(x)", Black, 25]}, AxesOrigin → {0, 0}]
```

Out[72]=

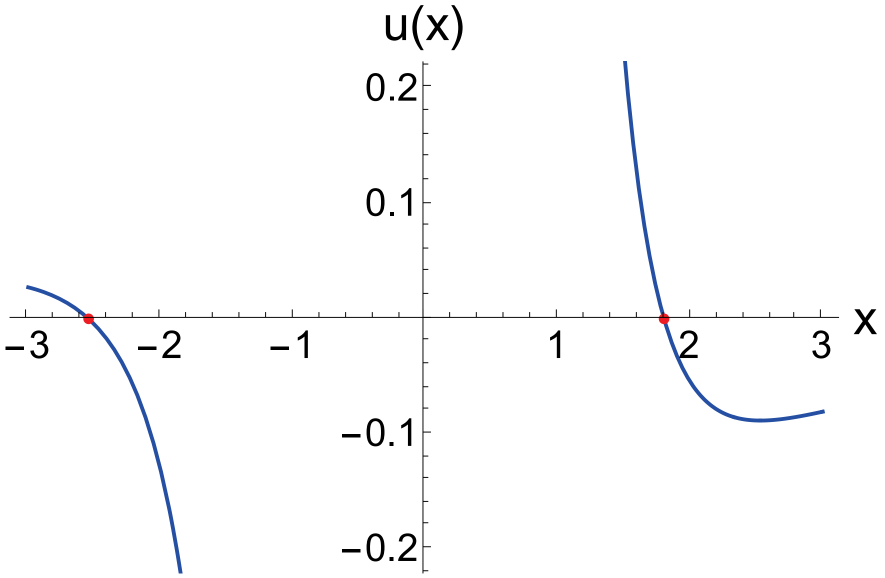

REPLICATING THE FAXEN ARGUMENT FOR THE ASYMMETRIC SWIMMER

Repeating the Faxen-inspired calculation for a swimmer dimer, we obtain the following separations. These are still symmetric, because while the interaction has an off-center nature to it, both swimmers would experience this.

In[73]:=
```
uaft = Simplify[utot /. {xf → x - z, yf → y}];
FaxAft = (uaft + (a^2 / 6) * (D[uaft, x, x] + D[uaft, y, y])) /. nsub;
ΔuAft = Simplify[FaxAft⟦1⟧ /. {y → 0, x → 0}];
```

In[76]:=
```
ufro = Simplify[utot /. {xf → x + z, yf → y}];
FaxFro = (ufro + (a^2 / 6) * (D[ufro, x, x] + D[ufro, y, y])) /. nsub;
ΔuFro = Simplify[FaxFro⟦1⟧ /. {y → 0, x → 0}];
```

In[79]:=
```
sol1 = FindRoot[(ΔuAft == ΔuFro), {z, -2}]
sol2 = FindRoot[(ΔuAft == ΔuFro), {z, 2}]
```

Out[79]= {z → -2.22755}

Out[80]= {z → 2.22755}

Plugging in the separation solution, we obtain a ‘speed up’ of -0.07. That is, the swimmer moves

faster along its direction of travel, which, we assigned to be along the negative x-axis, as was made clear in the above picture.

In[81]:=
```
ΔuFro /. sol1
ΔuAft /. sol2
```

Out[81]= -0.0701911

Out[82]= -0.0701911

Applying the same argument to a trimer, we find that the mirror symmetry in the contours providing both solution sets is lost. Consequently, the distance between the front and aft pairs is different.

In[83]:=
```
ufro =
  Simplify[utot /. {xf → x + r, yf → y}] + Simplify[utot /. {xf → x + (r + q), yf → y}];
FaxFro = (ufro + (a^2 / 6) * (D[ufro, x, x] + D[ufro, y, y])) /. nsub;
ΔuFro = Simplify[FaxFro[[1]] /. {y → 0, x → 0}];
```

In[86]:=
```
umid = Simplify[utot /. {xf → x - r, yf → y}] + Simplify[utot /. {xf → x + q, yf → y}];
FaxMid = (umid + (a^2 / 6) * (D[umid, x, x] + D[umid, y, y])) /. nsub;
ΔuMid = Simplify[FaxMid[[1]] /. {y → 0, x → 0}];
```

In[89]:=
```
uaft =
  Simplify[utot /. {xf → x - q, yf → y}] + Simplify[utot /. {xf → x - (q + r), yf → y}];
FaxAft = (uaft + (a^2 / 6) * (D[uaft, x, x] + D[uaft, y, y])) /. nsub;
ΔuAft = Simplify[FaxAft[[1]] /. {y → 0, x → 0}];
```

In[92]:=
```
pl0 = (ΔuFro - ΔuMid);
pl1 = (ΔuAft - ΔuMid);
Show[ContourPlot[pl0, {q, 1, 4}, {r, 1, 4}, Contours → {0},
   ContourStyle → {{Blue, Thick}}, ContourShading → None],
ContourPlot[pl1, {q, 1, 4}, {r, 1, 4}, Contours → {0},
   ContourStyle → {{Red, Thick}}, ContourShading → None],
 ImageSize → 500, FrameStyle → Directive[{Black, 20}],
 FrameLabel → {Style["Δx12", Black, 25], Style["Δx23", Black, 25]}]
```

Out[94]=

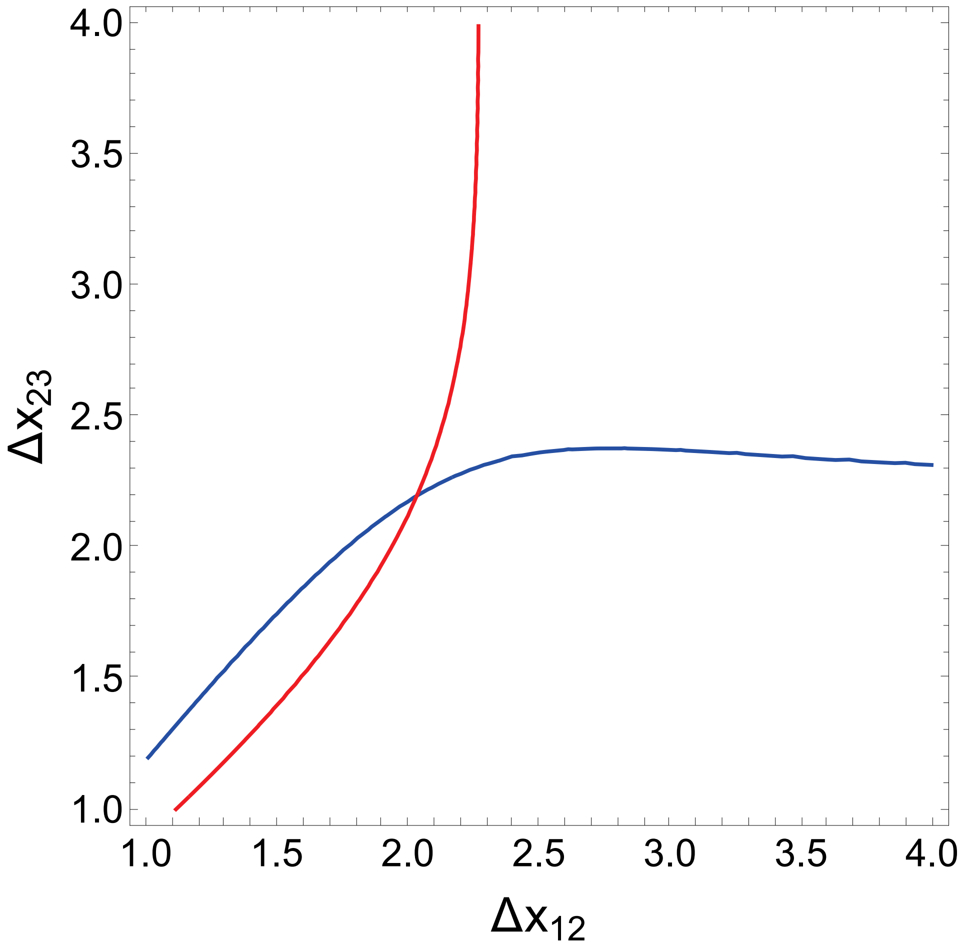

The front pair is slightly closer together than the aft pair.

In[95]:=
```
set = {ΔuFro == ΔuMid, ΔuMid == ΔuAft};
solset = FindRoot[set, {{q, 2, 1, 5}, {r, 2, 1, 5}}]
```

Out[96]= {q → 2.02904, r → 2.20017}

Contrasting with the dimer solution, we find that the overall spacing is somewhat reduced with the front pair having a separation of 91% of that of the dimer, while the aft pair has a separation of 99% of that of the dimer.

In[97]:=
```
-solset⟦1, 2⟧ / sol1⟦1, 2⟧
solset⟦2, 2⟧ / sol2⟦1, 2⟧
```

Out[97]= 0.910886

Out[98]= 0.987706

The associated speed-up is slightly greater than that for the dimer. Here it should be noted that we compare speed-up rather than absolute speed, which remains a free parameter.

In[99]:=
```
ΔuFro /. solset
```
Out[99]=
```
-0.118321
```

ESTIMATING THE PARAMETERS

In the experiment we find around a single obstacle of the same radius a dimer with a mean separation of 5.3 μm and a trimer with leading-pair separation 3.8 μm and trailing-pair separation 4.3 μm. The diameter of the swimmer is 2 μm, implying a reduced dimer separation of 2.65 and similarly reduced separations of 1.9 and 2.15 for the trimer. We can readily tune s and the ratio κ/λ to obtain the former.

In[100]:=
```
nsub = {a → 1 / 2, κ → 7.05, λ → 1, s → 0.7};
```
In[101]:=
```
uaft = Simplify[utot /. {xf → x - z, yf → y}];
FaxAft = (uaft + (a^2 / 6) * (D[uaft, x, x] + D[uaft, y, y])) /. nsub;
ΔuAft = Simplify[FaxAft[[1]] /. {y → 0, x → 0}];
```
In[104]:=
```
ufro = Simplify[utot /. {xf → x + z, yf → y}];
FaxFro = (ufro + (a^2 / 6) * (D[ufro, x, x] + D[ufro, y, y])) /. nsub;
ΔuFro = Simplify[FaxFro[[1]] /. {y → 0, x → 0}];
```
In[107]:=
```
sol1 = FindRoot[(ΔuAft == ΔuFro), {z, -2}];
sol2 = FindRoot[(ΔuAft == ΔuFro), {z, 2}]
```
Out[108]=
```
{z → 2.6505}
```

However the ratio for the trimer is not the same and cannot be obtained by varying s over a reasonable range. This is to be expected, as our approximation does not account for the size and presence of the post, or higher-order contributions to the flow fields. Nonetheless, a judicious choice of parameters shows that the trend can be qualitatively captured.

In[109]:=
```
ufro =
  Simplify[utot /. {xf → x + r, yf → y}] + Simplify[utot /. {xf → x + (r + q), yf → y}];
FaxFro = (ufro + (a^2 / 6) * (D[ufro, x, x] + D[ufro, y, y])) /. nsub;
ΔuFro = Simplify[FaxFro[[1]] /. {y → 0, x → 0}];
```
In[112]:=
```
umid = Simplify[utot /. {xf → x - r, yf → y}] + Simplify[utot /. {xf → x + q, yf → y}];
FaxMid = (umid + (a^2 / 6) * (D[umid, x, x] + D[umid, y, y])) /. nsub;
ΔuMid = Simplify[FaxMid[[1]] /. {y → 0, x → 0}];
```
In[115]:=
```
uaft =
  Simplify[utot /. {xf → x - q, yf → y}] + Simplify[utot /. {xf → x - (q + r), yf → y}];
FaxAft = (uaft + (a^2 / 6) * (D[uaft, x, x] + D[uaft, y, y])) /. nsub;
ΔuAft = Simplify[FaxAft[[1]] /. {y → 0, x → 0}];
```

```
In[118]:= pl0 = (ΔuFro - ΔuMid);
          pl1 = (ΔuAft - ΔuMid);
          set = {ΔuFro == ΔuMid, ΔuMid == ΔuAft};
          solset = FindRoot[set, {{q, 2, 1, 5}, {r, 2, 1, 5}}]
Out[121]= {q → 2.41056, r → 2.57402}
```

TRAIN OF SWIMMERS

Lastly, we examine what happens when we increase the train length. We go back to our original parameters here.

```
In[211]:= PlotSwim[x_] := Show[Graphics[
             {EdgeForm[{Thick, Black}], White, Disk[{x, 0}, 1 / 2, {Pi / 2, 3 * Pi / 2}]}],
            Graphics[{EdgeForm[{Thick, Gray}], Gray, Disk[{x, 0}, 1 / 2, {-Pi / 2, Pi / 2}]}],
            ImageSize → {Automatic, 50}]
          PlotSwim[
           0]
```

Out[212]=

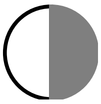

Dimer

The separation is visualized as follows:

```
In[203]:= S = 2;
          tU = Table[Sum[
               If[i == j, {0, 0}, utot /. {xf → x + (t[j] - t[i]), yf → y}], {j, 1, S}], {i, 1, S}];
          tF = Table[(tU⟦i⟧ + (a^2 / 6) * (D[tU⟦i⟧, x, x] + D[tU⟦i⟧, y, y])) /. nsub, {i, 1, S}];
          tD = Table[tF⟦i, 1⟧ /. {y → 0, x → 0}, {i, 1, S}];
          eqs = Table[(tD⟦i⟧ == tD⟦i + 1⟧) /. {t[S] → 0} /. nsub, {i, 1, S - 1}];
          trial = Table[{t[S - i], 2 * i + 1, 2 * i, 2 * i + 2}, {i, 1, S - 1}];
          sols = FindRoot[eqs, trial];
          Show[PlotSwim[0], Table[PlotSwim[sols⟦i, 2⟧], {i, 1, Length[sols]}],
           ImageSize → {Automatic, 50}]
```

Out[210]=

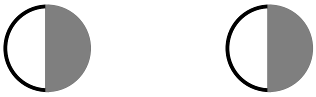

The separation is given by the ratio between the subsequent terms being (not defined for a dimer):

```
In[132]:= dists =
           Prepend[Table[sols⟦i + 1, 2⟧ - sols⟦i, 2⟧, {i, 1, Length[sols] - 1}], sols⟦1, 2⟧]
          Table[dists⟦i + 1⟧ / dists⟦1⟧, {i, 1, Length[dists] - 1}]
Out[132]= {2.6505}
Out[133]= {}
```

And finally the speed-up is given by:

In[134]:=
```
DeleteDuplicates[
  Round[Transpose[tF /. sols /. {t[S] → 0} /. nsub /. {y → 0, x → 0}][[1]],
   1.0 * 10 ^ (-6)]][[1]]
```

Out[134]= -0.0404

Trimer

The separation is visualized as follows:

In[187]:=
```
S = 3;
tU = Table[Sum[
    If[i == j, {0, 0}, utot /. {xf → x - (t[j] - t[i]), yf → y}], {j, 1, S}], {i, 1, S}];
tF = Table[(tU[[i]] + (a^2 / 6) * (D[tU[[i]], x, x] + D[tU[[i]], y, y])) /. nsub, {i, 1, S}];
tD = Table[tF[[i, 1]] /. {y → 0, x → 0}, {i, 1, S}];
eqs = Table[(tD[[i]] == tD[[i + 1]]) /. {t[S] → 0} /. nsub, {i, 1, S - 1}];
trial = Table[{t[S - i], 2 * i + 1, 2 * i, 2 * i + 2}, {i, 1, S - 1}];
sols = FindRoot[eqs, trial]
Show[PlotSwim[0], Table[PlotSwim[sols[[i, 2]]], {i, 1, Length[sols]}],
 ImageSize → {Automatic, 50}]
```

Out[193]= {t[2] → 2.41056, t[1] → 4.98458}

Out[194]= 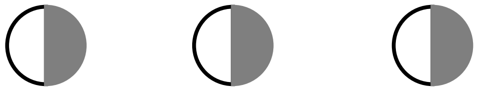

The separation is given by the ratio between the subsequent terms being:

In[143]:=
```
dists =
 Prepend[Table[sols[[i + 1, 2]] - sols[[i, 2]], {i, 1, Length[sols] - 1}], sols[[1, 2]]]
Table[dists[[i + 1]] / dists[[1]], {i, 1, Length[dists] - 1}]
```

Out[143]= {2.41056, 2.57402}

Out[144]= {1.06781}

And finally the speed-up is given by:

In[145]:=
```
DeleteDuplicates[
  Round[Transpose[tF /. sols /. {t[S] → 0} /. nsub /. {y → 0, x → 0}][[1]],
   1.0 * 10 ^ (-6)]][[1]]
```

Out[145]= -0.069742

Quadrumer

The separation is visualized as follows:

In[195]:=
```
S = 4;
tU = Table[Sum[
     If[i == j, {0, 0}, utot /. {xf → x - (t[j] - t[i]), yf → y}], {j, 1, S}], {i, 1, S}];
tF = Table[(tU⟦i⟧ + (a^2 / 6) * (D[tU⟦i⟧, x, x] + D[tU⟦i⟧, y, y])) /. nsub, {i, 1, S}];
tD = Table[tF⟦i, 1⟧ /. {y → 0, x → 0}, {i, 1, S}];
eqs = Table[(tD⟦i⟧ == tD⟦i + 1⟧) /. {t[S] → 0} /. nsub, {i, 1, S - 1}];
trial = Table[{t[S - i], 2 * i + 1, 2 * i, 2 * i + 2}, {i, 1, S - 1}];
sols = FindRoot[eqs, trial]
Show[PlotSwim[0], Table[PlotSwim[sols⟦i, 2⟧], {i, 1, Length[sols]}],
 ImageSize → {Automatic, 50}]
```

Out[201]= {t[3] → 2.30049, t[2] → 4.63262, t[1] → 7.23141}

Out[202]=

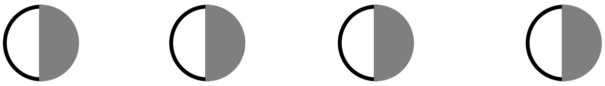

The separation is given by the ratio between the subsequent terms being:

In[154]:=
```
dists =
 Prepend[Table[sols⟦i + 1, 2⟧ - sols⟦i, 2⟧, {i, 1, Length[sols] - 1}], sols⟦1, 2⟧]
Table[dists⟦i + 1⟧ / dists⟦1⟧, {i, 1, Length[dists] - 1}]
```

Out[154]= {2.30049, 2.33213, 2.5988}

Out[155]= {1.01375, 1.12967}

And finally the speed-up is given by:

In[156]:=
```
DeleteDuplicates[
   Round[Transpose[tF /. sols /. {t[S] → 0} /. nsub /. {y → 0, x → 0}]⟦1⟧,
    1.0 * 10^(-6)]]⟦1⟧
```

Out[156]= -0.090964

Pentamer

The separation is visualized as follows:

In[179]:=
```
S = 5;
tU = Table[Sum[
     If[i == j, {0, 0}, utot /. {xf → x - (t[j] - t[i]), yf → y}], {j, 1, S}], {i, 1, S}];
tF = Table[(tU⟦i⟧ + (a^2 / 6) * (D[tU⟦i⟧, x, x] + D[tU⟦i⟧, y, y])) /. nsub, {i, 1, S}];
tD = Table[tF⟦i, 1⟧ /. {y → 0, x → 0}, {i, 1, S}];
eqs = Table[(tD⟦i⟧ == tD⟦i + 1⟧) /. {t[S] → 0} /. nsub, {i, 1, S - 1}];
trial = Table[{t[S - i], 2 * i + 1, 2 * i, 2 * i + 2}, {i, 1, S - 1}];
sols = FindRoot[eqs, trial]
Show[PlotSwim[0], Table[PlotSwim[sols⟦i, 2⟧], {i, 1, Length[sols]}],
 ImageSize → {Automatic, 50}]
```

Out[185]= {t[4] → 2.23891, t[3] → 4.46209, t[2] → 6.81818, t[1] → 9.48168}

Out[186]=

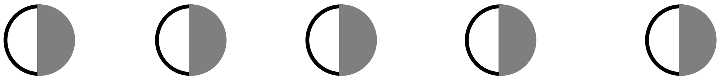

The separation is given by the ratio between the subsequent terms being:

In[165]:=
```
dists =
 Prepend[Table[sols⟦i + 1, 2⟧ - sols⟦i, 2⟧, {i, 1, Length[sols] - 1}], sols⟦1, 2⟧]
Table[dists⟦i + 1⟧ / dists⟦1⟧, {i, 1, Length[dists] - 1}]
```

Out[165]= {2.23891, 2.22318, 2.35609, 2.6635}

Out[166]= {0.992974, 1.05234, 1.18964}

And finally the speed-up is given by:

In[167]:=
```
DeleteDuplicates[
  Round[Transpose[tF /. sols /. {t[S] → 0} /. nsub /. {y → 0, x → 0}]⟦1⟧,
   1.0 * 10^(-6)]]⟦1⟧
```

Out[167]= -0.105695

Hexamer

The separation is visualized as follows:

In[168]:=
```
S = 6;
tU = Table[Sum[
     If[i == j, {0, 0}, utot /. {xf → x - (t[j] - t[i]), yf → y}], {j, 1, S}], {i, 1, S}];
tF = Table[(tU⟦i⟧ + (a^2 / 6) * (D[tU⟦i⟧, x, x] + D[tU⟦i⟧, y, y])) /. nsub, {i, 1, S}];
tD = Table[tF⟦i, 1⟧ /. {y → 0, x → 0}, {i, 1, S}];
eqs = Table[(tD⟦i⟧ == tD⟦i + 1⟧) /. {t[S] → 0} /. nsub, {i, 1, S - 1}];
trial = Table[{t[S - i], 2 * i + 1, 2 * i, 2 * i + 2}, {i, 1, S - 1}];
sols = FindRoot[eqs, trial]
Show[PlotSwim[0], Table[PlotSwim[sols⟦i, 2⟧], {i, 1, Length[sols]}],
 ImageSize → {Automatic, 50}]
```

Out[174]= {t[5] → 2.2026, t[4] → 4.36714, t[3] → 6.61708, t[2] → 9.04004, t[1] → 11.7901}

Out[175]=

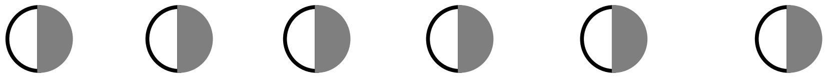

The separation is given by the ratio between the subsequent terms being:

In[176]:=
```
dists =
 Prepend[Table[sols⟦i + 1, 2⟧ - sols⟦i, 2⟧, {i, 1, Length[sols] - 1}], sols⟦1, 2⟧]
Table[dists⟦i + 1⟧ / dists⟦1⟧, {i, 1, Length[dists] - 1}]
```

Out[176]= {2.2026, 2.16454, 2.24994, 2.42296, 2.75005}

Out[177]= {0.982721, 1.02149, 1.10005, 1.24855}

And finally the speed-up is given by:

In[178]:=
```
DeleteDuplicates[
  Round[Transpose[tF /. sols /. {t[S] → 0} /. nsub /. {y → 0, x → 0}]⟦1⟧,
   1.0 * 10^(-6)]]⟦1⟧
```

Out[178]= -0.115028

Conclusion

A simple, physics-motivated model can capture the experimentally observed speed-up of trains of swimmers and heterogeneity in such a train. Note, however, that this is a simple model that is aimed at providing a possible explanation. The authors do not claim that this is the only way the experimental observations can be explained.
