# Supplementary material for: Activity-induced interactions and cooperation of artificial microswimmers in one-dimensional environments
Source: Nat Commun. 2022 Apr 1;13:1772. doi: 10.1038/s41467-022-29430-1 (PMC8976030; doi:10.1038/s41467-022-29430-1)
Supplement: Supplementary file 2 — Description of SI files [file 41467_2022_29430_MOESM2_ESM.pdf]

Description of Additional Supplementary Files for: Activity-induced microswimmer interactions and cooperation in one-dimensional environments

Stefania Ketzetzi, Melissa Rinaldin, Pim Dröge, Joost de Graaf,

Daniela J. Kraft

File Name: Supplementary Movie 1
Description: Swimmers that orbit the post with opposing direction of motion become immobilized on the post. The movie shows two catalytic swimmers of 2 µm diameter and 4.7 nm Pt that remain stationary in close contact along a circular post of 12 µm diameter (duration ≈ 1 min, real time, 5 fps).

File Name: Supplementary Movie 2
Description: The movie shows two catalytic swimmers of 2 µm diameter and 4.7 nm Pt orbiting a circular post of diameter 4 µm (duration ≈ 1 min, real time, 5 fps). Scale bar is 5 µm.

File Name: Supplementary Movie 3
Description: The movie shows three catalytic swimmers of 2 µm diameter and 4.7 nm Pt orbiting a circular post of diameter 4 µm (duration ≈ 1 min, real time, 5 fps). Scale bar is 5 µm.

File Name: Supplementary Movie 4
Description: The movie shows two catalytic swimmers of 2 µm diameter and 4.7 nm Pt orbiting a circular post of diameter 8 µm (duration ≈ 1 min, real time, 5 fps). Scale bar is 5 µm.

File Name: Supplementary Movie 5
Description: The movie shows the dynamic formation of a microswimmer chain (duration 9 s, real time, 9 fps). The 2 µm diameter swimmers initially move clockwise in a train along a peanut-shaped post. After encountering an immobile cluster at the edge, they compact into a long self-propelling chain.

File Name: Supplementary Movie 6
Description: Necks with high curvature act as accumulation sites for the 2 µm diameter swimmers. The movie shows the formation of a stationary microswimmer chain along the top neck side of a peanut-shaped post with high neck curvature,

while a formerly assembled chain also remains at rest along the bottom neck side of the post (duration 24 s, real time, 9 fps).

File Name: Supplementary Movie 7
Description: The movie shows an actively-assembled microswimmer chain self- propelling along a peanut-shaped post (duration 32 s, real time, 9 fps). During orbiting, swimmers escape the chain from the back of the chain when they reach the rounded post edge, and from the middle of the chain when they reach the neck or the rounded post edge.

File Name: Supplementary Movie 8
Description: The movie shows an example of a microswimmer chain breakup into two parts along a peanut-shaped post, followed by remobilization of one of the parts and subsequent reformation of the entire chain at the neck site of the post (duration 38 s, real time, 9 fps).

File Name: Supplementary Software
Description: Mathematica notebook containing calculations on the collective effects of co-moving microswimmers.
